# Elite Bernoulli-based mutated dung beetle algorithm for global complex problems and parameter estimation of solar photovoltaic models

**DOI:** 10.1038/s41598-025-06108-4

**Published:** 2025-10-17

**Authors:** Mohamed Elhosseny, Mahmoud Abdel-Salam, Anand Nayyar, Emre Çelik, Shubham Mahajan, Nebojsa Bacanin, Ibrahim M. El-Hasnony

**Affiliations:** 1 College of Computing and Informatics, University of Sharjah, Sharjah, UAE; 2 Faculty of Computer and Information Sciences, Mansoura University, Mansoura, 35516 Egypt; 3 School of Computer Science, Duy Tan University, Da Nang, 550000 Viet Nam; 4 Department of Electrical and Electronics Engineering, Faculty of Engineering, Düzce University, Düzce, Turkey; 5 Amity School of Engineering & Technology, Amity University Haryana, Gurugram, India; 6 Faculty of Informatics and Computing, Singidunum University, Danijelova 32, 11000 Belgrade, Serbia; 7 Department of Mathematics, Saveetha School of Engineering, SIMATS, Thandalam, Chennai, Tamilnadu 602105 India

**Keywords:** Bernoulli operator, Morlet wavelet, Dung beetle optimization algorithm, Solar photovoltaic parameter estimation, Global optimization, Energy science and technology, Engineering, Civil engineering, Electrical and electronic engineering, Energy infrastructure, Mechanical engineering

## Abstract

The Dung Beetle Optimization (DBO) algorithm is a relatively recent metaheuristic known for its simplicity, versatility, and low parameter dependence, making it a valuable tool for solving complex optimization problems. Despite its potential, DBO suffers from limitations such as slow convergence and premature stagnation in local optima. To address these critical issues, this paper introduces a novel enhanced variant named Elite Bernoulli-based Mutated Dung Beetle Optimizer with Local Escaping Operator (EBMLO-DBO), specifically designed to improve the convergence speed, search capability, and robustness of the original DBO algorithm. The motivation for this enhancement stems from DBO’s limited performance in high-dimensional and non-convex problems, where it often fails to maintain an effective balance between exploration and exploitation. The novelty of the proposed EBMLO-DBO lies in the integration of four key strategies tailored to overcome these weaknesses: (i) Bernoulli map-based initialization to enhance population diversity and ensure a better global search foundation; (ii) Morlet Wavelet mutation to introduce adaptive local refinements and help the algorithm escape local optima; (iii) elite guidance to accelerate convergence by directing the population toward high-quality regions; and (iv) a local escaping operator (LEO) to dynamically refine the search process and strengthen exploitation without sacrificing exploration. The performance of EBMLO-DBO is rigorously validated using the CEC2017 and CEC2022 benchmark suites, where it achieves Friedman ranks of 1.83 and 2.7 respectively, consistently surpassing eleven state-of-the-art algorithms including PSO, HHO, WOA, and advanced methods like CMAES and IMODE. In benchmark function optimization, EBMLO-DBO demonstrates superior performance by achieving first rank in 50% of CEC2022 functions and obtaining the lowest average fitness values in 18 out of 29 CEC2017 functions. For photovoltaic parameter estimation applications, EBMLO-DBO exhibits exceptional accuracy with RMSE values of 9.8602E-4 for single diode models, 9.81307E-4 for double diode models, and 2.32066E-3 for PV module models, achieving top performance ranks of 1.45, 1.42, and 1.74, respectively. Statistical analysis using Wilcoxon signed-rank test at significance level $\alpha =0.05$ confirms the significant superiority of EBMLO-DBO over all compared algorithms, thereby validating the effectiveness and reliability of the proposed enhancements. Overall, the results state that EBMLO-DBO offers a significantly improved search performance and solution quality compared to the original DBO and related methods, thereby justifying the necessity and effectiveness of the proposed enhancements.

## Introduction

Global optimization has become a necessary component in various scientific disciplines in recent years. The real-world problems’ complexity has increased in recent decades, raising significant concerns in the optimization field. It is possible to view almost all issues in the actual world as optimization problems. The purpose of high-performance algorithms is to manage potentially difficult problems. Consequently, scientists are trying to create effective optimization strategies for these types of issues. Many optimization techniques have been introduced and modified to provide the best solutions for a range of problems in a variety of fields, including economics, engineering, machine learning, energy, medical, networks, and more^1,2^. According to the characteristics of their behaviors, these algorithms can be categorized as either metaheuristic or deterministic^3^. Within deterministic methodologies, the solution obtained from the preceding iteration is employed to deduce the revised solutions for the present iteration. The initial solution may affect the use of deterministic methods, which may affect the result. Furthermore, the search space gradient might be required in some circumstances. Besides, the solutions may converge to local optima^4^. Hard optimization problems pose serious obstacles in front of the deterministic methods which provides comparatively low success and efficiency rates. Therefore, new methodologies must be developed for optimization capable of determining near-optimal solutions when only limited computational effort is available. Over the last twenty years there has been an ever-increasing interest by the researchers in applying metaheuristic algorithms (MAs) to solve real-world problems^5,6^. Ease and simplicity, ease of usage, flexibility, and effective efficiency in computation in their application have attracted the interest of researchers towards the application of MAs for solving practical issues^7^. Also, since MAs do not rely on any operations involving gradients, it can work with less memory. It has the ability to quickly and simply address a variety of problems^8^.

It follows that the categories of MAs are human-activities based algorithms, natural law-based algorithms, evolution-based algorithms, and group-intelligence based algorithms^9,10^. Evolutionary algorithms are Genetic Algorithm (GA)^11^, for instance, quantum evolutionary algorithm (QEA)^12^, and the evolutionary programming (EP)^13^. Group-intelligence based algorithms are simulated on the routines of various natural swarming species. For instance, the Particle Swarm Algorithm (PSO)^14^, the Secretary Bird Optimizer (SBOA)^15^, the Nutcracker Optimizer (NOA)^16^, the Sled Dog Optimizer (SDO)^17^, the Coati Optimization Algorithm (COA)^18^, the Snake Optimizer (SO)^19^, the Crayfish Optimization Algorithm^20^, the Parrot Optimizer^21^, the Elk Herd Optimizer^22^, the Dung Beetle Optimizer (DBO)^23^, and the Sea Horse Optimization Algorithm (SHO)^24^, among others.

Furthermore, the third category of algorithms can be categorized into physics, and mathematical algorithms. The mathematical inspired algorithms include Sinh-Cosh algorithm^25^, runge kutta optimization algorithm (RUN)^26^, the Triangulation Topology Aggregation Optimizer (TTAO)^27^, and Newton Raphson Optimizer (NRO)^28^. In addition, the physics-based algorithms include Polar Lights Optimizer (PLO)^29^, Kepler law optimizer (KOA)^30^, geyser inspired algorithm^31^, lightning search algorithm (LSA)^32^, Fick’s Law optimizer (FLA)^33^. A group of intelligent algorithms has also been proposed that are based on the activities of human beings. These include the political optimizer (PO)^34^, the Poor and rich optimization algorithm (PRO)^35^, Human memory search algorithm (HMO)^36^, Human evolutionary algorithm (HEO)^37^, the Mother optimization algorithm (MOA)^38^ and the Skill optimizer (SOA)^39^ and etc.

Since MAs have efficient and flexible computational performance, they are frequently applied in practical optimization scenarios across a wide range of fields^40–43^. In recent years, the expansion of numerous disciplines and the advancement of technology and modern science have led to the emergence of complex, non-convex and large-scale problems^44^. While many intelligent algorithms have proven to be more effective than one another in cracking actual optimization issues, they still encounter challenges such as inadequate convergence, limited exploration capability, and performance decline when confronted with increasingly difficult and intricate problems across different fields^45,46^. Furthermore, the "No Free Lunch" (NFL) theory asserts that there is no algorithm now in use that can consistently outperform all other algorithms in any optimization situation that may arise^47^. Based on this investigation, it is critical to investigate quick and effective solutions for real-world application instances. Thus, it is necessary to continuously improve and enhance the algorithms’ performance in order to deliver more precise and ideal solutions to actual optimization scenarios.

On the other hand, the Dung Beetle Optimizer (DBO) is a new MA that was presented in 2023 and relies on the population notion to solve multiple problems^23^. The foraging habits of dung beetles served as the model for the DBO algorithm^23^. The DBO has fewer parameters and is much more resilient than other MAs^48^. The primary motivation for developing a novel EBMLO-DBO stems from critical limitations observed in current MAs, particularly the recently proposed DBO, which significantly impact their practical applicability in complex engineering problems. Despite DBO’s demonstrated efficiency in basic optimization scenarios, four fundamental weaknesses limit its effectiveness in high-dimensional and non-convex optimization problems. First, premature convergence occurs when dealing with complex multimodal landscapes, leading to suboptimal solutions and inadequate exploration of the search space, particularly evident in problems with numerous local optima. Second, existing algorithms including DBO fail to maintain an effective balance between exploration and exploitation phases, often either getting trapped in local regions or spending excessive computational resources on unnecessary exploration. Third, conventional initialization strategies rely heavily on random generation, which may not provide sufficient population diversity for comprehensive search space exploration, limiting the algorithm’s ability to locate promising regions efficiently. Fourth, the absence of effective local optima escape mechanisms restricts the algorithm’s capability to find global optima in challenging optimization scenarios, particularly in real-world applications like photovoltaic parameter estimation where high precision and reliability are crucial for practical implementation. Moreover, the "No Free Lunch" (NFL) theorem states that it is impossible for any MA to handle all optimization problems^47^. This implies that while the MA may provide good solutions for certain optimization problems, it may perform poorly for other problems. Therefore, appropriate, and efficient solutions are required to enhance the algorithm’s efficiency. Also, researchers are continuously devising novel and efficient algorithms as well as refining already-existing optimization algorithms to better tackle real-world engineering optimization problems. Consequently, pertinent literature is required to examine and enhance DBO. These justifications drive the improvement of a new Dung beetle optimization algorithm in this study. These limitations directly impact the performance and applicability of optimization algorithms in engineering applications, motivating the systematic development of EBMLO-DBO with targeted enhancement strategies designed to address each identified weakness comprehensively.

According to these motivations, this paper presents the enhanced Dung beetle optimization algorithm EBMLO-DBO with stronger performance, based on the aforementioned motivation. It does this by integrating four primary strategies including the Bernoulli search map, Elite guidance strategy, mixed mutation strategy and Local escape operator into the original DBO algorithm. The Bernoulli map is used to provide diverse population-based agents at the initialization phase which provide more away regions into the race of search process. Like many other MA, DBO agents are susceptible to be trapped in local solutions which affect the search ability of DBO, so Morlet mutation is presented to mutate the population and provide mutated enhanced agents to escape from the local optima. The majority of the MA’s processes are exploration and exploitation; the elite guidance strategy can enhance the quality of solutions presented and enrich the population’s exploration routes before iteration begins. As iteration progresses, the exploitation operator gradually gains the upper hand, improving the efficiency of global exploration. Furthermore, we present the local escape operator is used to enhance the exploitation ability of DBO and provide more accurate and high-quality solutions for the next generations which boosts the convergence speed at early iterations and remove the detrimental effect of invalid solutions. This study evaluates the enhanced EBMLO-DBO algorithm in comparison to a variety of advanced and traditional algorithms on different test sets CEC’17 and CEC’22. The statistical analysis is conducted with the Friedman rank and Wilcoxon rank sum tests. The results indicate a substantial enhancement in the performance of the DBO algorithm, and EBMLO-DBO shows notable competitiveness in comparison to other methods. Furthermore, the enhanced EBMLO-DBO method is utilized to address a practical issue of estimating the parameters of three photovoltaic (PV) models, which consist of single and double diode as well as PV module. Moreover, the EBMLO-DBO algorithm is more stable in comparison to most algorithms; thus, the solutions present higher accuracy and are likely to provide a suitable development methodology.

### Objectives of the paper

The objectives of the paper are:

1. To develop EBMLO-DBO algorithm by integrating four novel enhancement strategies: Bernoulli map-based initialization, Elite Leadership Strategy, Morlet Wavelet mutation, and Local Escaping Operator to overcome DBO’s limitations in high-dimensional optimization problems;
2. To integrate Bernoulli map that replaces conventional random initialization with chaotic sequences providing superior search space coverage, while the Elite Leadership Strategy dynamically adjusts selection probability between elite random leadership and best leadership to maintain optimal exploration–exploitation balance throughout optimization;
3. The proposed mutation strategy dynamically adjusts mutation space using iteration-dependent scaling parameters, providing strong exploration in early stages and fine-tuning capabilities in later optimization phases. The utilization of the Local Escaping Operator (LEO) to enhance solution quality and accelerate convergence through strategic position updates based on elite guidance;
4. And, to demonstrate practical engineering applicability of EBMLO-DBO through successful implementation in photovoltaic parameter estimation problems for single diode, double diode, and PV module models, establishing the algorithm’s real-world value in renewable energy system optimization and parameter identification applications.

### Organization of paper

The rest of the paper is organized as: Related works to the proposed algorithm are presented in Section "Literature review". The mathematical model and the main description of the original DBO algorithm is presented and discussed in Section "Related terminologies". Section "Proposed methodology: EBMLO-DBO algorithm for global complex problem and parameter estimation of solar photovoltaic model" presents the main proposed improvements integrated with DBO to enhance and boost its convergence and solution quality. Section "Experimental results" displays the conducted experiments for EBMLO-DBO using global optimization sets of functions while Section "PV parameter estimation using EBMLO-DBO" test and evaluate the EBMLO-DBO for solving a real problem for estimating the parameters of three PV models. The main discussions related to the overall presented experiments are presented in Section "Discussion". And, finally, section "Conclusion and future scope" concludes the paper with future scope.

## Literature review

In recent years, the field of PV system optimization has seen significant advancements through the development and application of various MAs. These approaches have been pivotal in addressing the challenges of parameter estimation and optimization in PV models, leading to improvements in accuracy, convergence, and robustness. This section summarizes key contributions from the literature related to the optimization problems and PV models, providing a foundation for identifying the research gaps addressed by our proposed work.

Zhu et al.^49^ proposed a multi-strategy DBO algorithm that integrated both quantum computing and multi-strategy fusion and was effective with a number of complex engineering problems. Mai et al.^50^ adopted the advanced variant of DBO to optimize wind speed forecasting, outperforming many other techniques such as inverse learning and Fuch chaotic functions.

Li et al.^51^ added a new version of DBO to optimize the parameters of LSTM models for identification of concrete dynamic principles, including integration with some novel methods like the greedy lens imaging back-learned one. The DBO algorithm with OBL improved by Zilong and Peng^52^ significantly outperformed the results on CEC’17 benchmarks and other engineering problems.

Zhong et al.^53^ proposed an enhanced variant of the Sine Cosine Algorithm by introducing a hierarchical multi-leadership search strategy to address limitations in population diversity and exploration–exploitation balance, enabling parallel guidance across multiple search trajectories. The algorithm demonstrated superior optimization performance across 18 classical benchmarks and 30 CEC’17 functions, consistently surpassing other state-of-the-art MAs. HMLSCA was also successfully applied to optimize SVM parameters and feature weights for medicine data classification across eight datasets, achieving the top Friedman rank of 1.00, and attained a 98% accuracy rate in COVID-19 diagnosis, validating its effectiveness in real-world applications.

Parizi et al.^54^ presented a hybrid MA approach by combining the exploration strength of the Woodpecker Mating Algorithm with the exploitation efficiency of the Sine Cosine Algorithm, enhanced further through Levy flight-based modifications and a shared local memory mechanism to dynamically balance search behaviors. The algorithm was validated on 28 complex benchmark functions, where it consistently outperformed recent metaheuristic algorithms in solving nonconvex and inseparable optimization tasks. Additionally, HSCWMA was applied as a trainer for Multi-Layer Perceptron networks in software development effort estimation problems, achieving superior results across three real-world datasets, demonstrating its adaptability and high-performance potential.

Karimzadeh Parizi et al.^55^ introduced an enhanced version of the Woodpecker Mating Algorithm by incorporating three key mechanisms: Distance Opposition-Based Learning (DOBL) for improved exploration and diversity, a local memory strategy to enhance exploitation, and a self-adaptive adjustment of the $H\alpha$ parameter to optimize the Running Away function’s effectiveness. The algorithm demonstrated strong performance across 23 benchmark functions, surpassing recent metaheuristic techniques in terms of convergence reliability and search efficiency. Additionally, OWMA was successfully applied as a trainer for Multi-Layer Perceptron neural networks on multiple biomedical and regression tasks, and further validated on five real-world constrained optimization problems, confirming its robustness and versatility in complex scenarios.

Xiao et al.^56^ proposed an advanced version of the Snow Ablation Optimizer by incorporating Good Point Set initialization, greedy selection, Differential Evolution-based search, and Dynamic Lens Opposition-Based Learning to overcome premature convergence, enhance diversity, and improve accuracy in complex high-dimensional problems. Extensive comparisons on the IEEE CEC2017 and CEC2022 benchmark functions showed that MSAO outperformed classical SAO and several leading algorithms, achieving the best overall performance with Friedman mean rankings of 1.66 and 1.25, respectively. Moreover, the proposed MSAO demonstrated strong practical effectiveness in solving six constrained engineering design problems and a photovoltaic model parameter estimation case, confirming its broad applicability and high optimization capability^57^.

A guaranteed convergence arithmetic optimization algorithm combined with a modified Newton–Raphson technique, GCAOAEmNR, for parameter optimization of PV models, was proposed by Ridha et al.^58^, indicating very high accuracy and stability under various conditions. Similarly, El-Dabah et al.^59^ used the Northern Goshawk Optimization algorithm for parameter estimation in triple diode PV models. The results demonstrated increased convergence speed and improved solution accuracy when it was applied to a few commercial PV modules.

Abbassi et al.^60^, presented an enhanced Arithmetic Optimization Algorithm (AOA) elaborated for parameter extraction in photovoltaic models, offering better applicability over varied conditions. The approach was applied to single and double diode PV models and gave results superior to other algorithms that already exist.

Long et al.^61^ presented the hybrid Seagull Optimization Algorithm for estimating PV parameters, much superior to the available algorithms, since it exhibits better precision and the power to avoid local optima. Janamala and Radha Rani^62^ presented an Archimedes Optimization Algorithm for optimal placement of solar photovoltaic with minimum grid dependence and improved voltage profiles.

In this respect, Lu et al., in^63^, proposed the HMSCPSO algorithm to enhance global search and avoid a possible local optima for an improved accuracy of PV model parameter estimation. Another option was NOA algorithm proposed by Duan et al.^64^ for extracting the PV parameters that present improved results against PSO, FWA, and WOA in terms of accuracy and efficiency of the parameter extraction process.

Ramadan et al. in^65^ presented the Enhanced Harris Hawk Optimization (EHHO) algorithm used to optimize PV model parameters by enriching the search phase through the incorporation of sine and cosine functions, leading to improved accuracy for multiple PV models. Additionally, Yousri et al.^66^ developed an Adaptive Fractional-order Archimedes Optimization Algorithm (A-FAOA), which outperformed state-of-the-art techniques for finding the best PV parameters under different scenarios.

Mohamed et al.^67^ proposed a Hybrid Kepler Optimization Algorithm (HKOA) for enhancing parameter estimation within PV models, while being able to balance exploration and exploitation effectively, outperforming existing advanced methodologies in the process. Kullampalayam Murugaiyan et al.^68^ introduced the Opposition-Based Exponential Distribution Optimizer (OBEDO) applied for photovoltaic parameters extraction which enhanced computational efficiency and accuracy.

A better algorithm in PV model parameter extraction was presented by Izci et al.^69^, which combines random learning with a logarithmic spiral search algorithm, and it was called En-PDO. A better accuracy in the estimation of the PV parameter was shown by Saadaoui et al.^70^ by combining BSA with DE.

Ekinci et al.^71^ proposed a hybrid Gazelle-Nelder-Mead algorithm for PV parameter extraction, showing its convergence and accuracy for the superiority of many benchmark tests. Qaraad et al.,^72^, proposed the QPSOL technique, based on PSO, increasing accuracy and efficiency of solution in the estimation of PV parameters.

Javed et al.^73^ suggested MPPT-based Flying Squirrel Search Optimization for hybrid PV-TEG systems. The algorithm provided very good efficiency and fast response in tracking. On the other hand, Kahraman et al.^74^ introduced a dynamic-fitness-distance-balance technique into the Stochastic Fractal Search algorithm, which led to increased accuracy and robustness in the estimation of photovoltaic parameters. Izci et al.^75^ developed a pattern search algorithm that would overcome the Mountain Gazelle Optimizer problems regarding low parameter estimation accuracy and reliability.

While important contributions have been made in putting metaheuristic algorithms to the estimation of PV parameters, no solid trade-off has been obtained so far between global exploration and local exploitation, especially in what concerns DBO variants. The proposed contributions always provide less-than-satisfactory convergence speed and accuracy, with inconsistent performance across different PV models. The proposed work aims to address these gaps by introducing a novel DBO variant (EBMLO-DBO) designed to enhance global optimization and parameter estimation accuracy in single, double, and PV models, thereby improving the overall efficiency and reliability of PV system modeling.

## Related terminologies

This section gives definitions to the most important terms connected with the proposed work as well as provides a broad overview of the original DBO algorithm and the reason why and how PV model parameters are determined in this procedure.

### Dung beetle algorithm (DBO)

A new algorithm called the DBO was presented in 2023^23^. Its goal is to find the best solutions through a variety of activities like ball rolling, dancing, foraging, breeding, and thieving. Therefore, there are four stages in this algorithm: foraging, breeding, rolling balls, and stealing.

#### Population initialization

Like any swarm-based optimization algorithm, DBO’s first phase is the population initialization which comprises the set of agents/ solutions used to crack the optimization problem. Random initialization is used in the original DBO to initialize the values of starting solutions in DBO. For a set of $N$ solutions and each solutions comprises a set of $D$ dimensions that represent the dimensionality of the cracked problem, the initialization phase can be represented as follows:

1 $${X}_{i}=Lb+rand()\times (Lb-Ub)$$

where the solution within the population is denoted by ${X}_{i}$ and the limits for the search pace of the cracked problem are defined by $Lb$ and $Ub$. In addition, a random value is generated between 0 and 1 using the built-in function $rand()$

#### Roller-ball dung beetle

In its native habitat, the dung beetle relies on the position of the sun to maintain a straight course when rolling a ball of excrement. During the rolling of the dung ball, the location of the dung beetle was updated using Eq. (2):

2 $$\begin{array}{cc}& {X}_{i}(t+1)={X}_{i}(t)+\alpha \cdot k\cdot {X}_{i}(t-1)+b\cdot \Delta x\\ & \Delta x=\left|{X}_{i}(t)-{X}^{w}\right|\end{array}$$

The current iteration and the current position of the dung beetle are represented by *t* and ${X}_{i}(t)$, respectively, in accordance with Eq. (2). The dung beetle’s divergence from the average direction is defined by the parameter $\alpha$. It accepts two random numbers, 1 and -1, where 1 denotes no deviation and -1 denotes a deviation. The defect factor, with a default value of 0.1, is defined by the variable $k$, which ranges from 0 to 0.2. It is assumed that the constant $b$ has a value of 0.3 and is restricted to the interval [0, 1]. Since the simulation of solar illumination is described by $\Delta x$ and the worst global value is ${X}^{w}$, a higher $\Delta x$ value would indicate a greater distance from the light source. To simulate the potential of hitting an obstruction while rolling a ball, an obstacle-hitting event is used. To mimic the dancing behavior of dung beetles, the tangent function is utilized to determine a new path for the ball to roll in the event of an impediment. Consequently, Eq. (3) describes the rolling beetle’s position:

3 $${X}_{i}(t+1)={X}_{i}(t)+tan(\theta )\left|{X}_{i}(t)-{X}_{i}(t-1)\right|$$

The range of 0 to $\pi$ encompasses the parameter θ and the position is not updated when it takes the values 0, $\pi /2$, and $\pi$ because there is no position change.

#### Spawning dung beetles

Dung beetles typically reproduce in safe places based on their natural surroundings. This approach, which is described as in Eq. (4), takes into account the selection of safe borders in the surroundings to symbolize secure zones:

4 $$\begin{array}{cc}& L{b}^{*}=max\left({X}^{*}\times (1-R),Lb\right)\\ & U{b}^{*}=min\left({X}^{*}\times (1+R),Ub\right)\end{array}$$

The variables $L{b}^{*}$ and $U{b}^{*}$, which define the lower and upper bounds, respectively, define the spawning region limits. The current local optimum is indicated by the variable ${X}^{*}$. The convergence parameter $R$ is defined as $1-t/T$, where $T$ is the maximum number of iterations. The dung beetle will therefore choose where to lay its egg within this predetermined range when it discovers the ideal location for breeding. In order to prevent the search from becoming isolated while trying to locate the current best solution, the spawning area is dynamically set so that the best position remains within the range of interest. The spawning beetle’s position update is explained as follows:

5 $${X}_{i}(t+1)={X}^{*}+{b}_{1}\times \left({X}_{i}(t)-L{b}^{*}\right)+{b}_{2}\times \left({X}_{i}(t)-U{b}^{*}\right)$$

The two randomly generated vectors, ${b}_{1}$ and ${b}_{2}$, each have a size of $1\times D$, where $D$ is the size of the optimization issue.

#### Foraging dung beetles

In their natural environment, the dung beetles forage in close proximity to the designated region when they choose a safe spot, which is close to their egg-laying process. The original study suggests the following formula for a new definition of this area:

6 $$\begin{array}{cc}& L{b}^{b}=max\left({X}^{b}\times (1-R),Lb\right)\\ & U{b}^{b}=min\left({X}^{b}\times (1+R),Ub\right)\end{array}$$

From this angle, ${X}^{b}$ would represent the ideal global position. The boundaries of the foraging area are denoted by $L{b}^{b}$ and $U{b}^{b}$, respectively. Furthermore, the range of the problem-solving domain is represented by $Lb$ and $Ub$. Equation (7) describes how the foraging dung beetle updates its position each time it engages in foraging behavior as follows:

7 $${X}_{i}(t+1)={X}_{i}(t)+{C}_{1}\times \left({X}_{i}(t)-L{b}^{b}\right)+{C}_{2}\times \left({X}_{i}(t)-U{b}^{b}\right)$$

where ${C}_{1}$ has a normal distribution and ${C}_{2}$ is a vector of size $1\times D$, and $D$ is the dimensionality of the problem and whose elements are between 0 and 1.

#### Stealing dung beetles

In their natural habitat, dung beetles on locations are not directly taken from other dung beetles; instead, they move one another in a competition for ownership. As a result, the authors estimated this behavior in the initial search using the best global location, or ${X}^{b}$. The thieving dung beetle repositions itself in such a way that Eq. (8) specifies the location update:

8 $${X}_{i}(t+1)={X}^{b}+S\cdot g\cdot \left(\left|{X}_{i}(t)-{X}^{*}\right|+\left|{X}_{i}(t)-{X}^{b}\right|\right)$$

Where $g$ be the random variable’s magnitude and let $S$ be a constant with a fixed value of 0.5 in Eq. (8). Six dung beetles are rolling dung, seven are foraging, eleven are thieving, and six are reproducing, according to the original paper.

### PV parameter estimation problem

Determining parameters in PV models is typically approached as an optimization problem aimed at minimizing the disparity between simulated results from estimated parameters and experimental data^76^. The objective of this optimization is to quantify the discrepancy between the current reported by the manufacturer and predicted current by the model. Various algorithms are utilized to find parameter sets that reduce this difference. Solar cell modeling involves three main steps: selecting appropriate equivalent models, developing mathematical models, and accurately determining cell parameter values. Firstly, identifying the electrical characteristics of a PV system is to carefully select the appropriate models. The double-diode and single-diode models are used to define the electrical properties of PV modules and cells^77^.

**Fig. 1:**
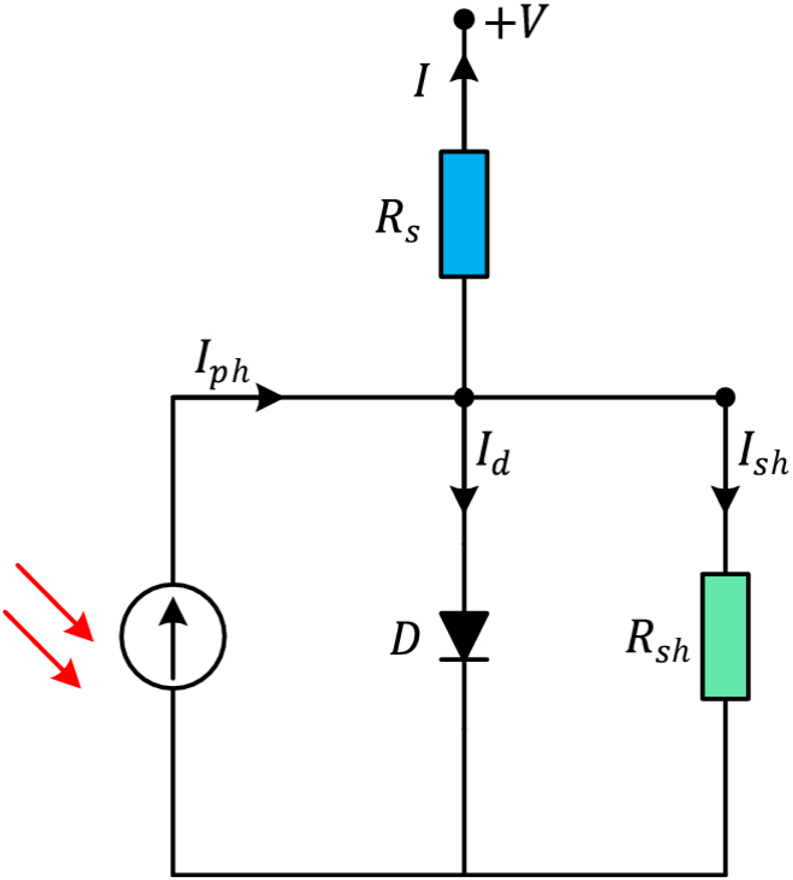
Circuit of Single diode model.

- The single diode is shown in Fig. 1. This model consists of five fundamental parameters: the ${I}_{rsD}$ which represents the diode reverse saturation current, ${I}_{PC}$ that represents the photovoltaic current, ${R}_{se}$, ${R}_{SH}$ which are the series and shunt resistances, respectively and $N$ denotes the ideality factor. Figure 1 depicts the lighted solar cell as a composite of two resistors, a diode and the current source. The series resistor symbolizes the resistance that is faced in the route of the current, whereas the leakage current is demonstrated by the shunt resistor. The series resistance comprises the contact resistance, electrode resistance, and resistance from the material itself. The output current ${I}_{out}$ can be calculated using Kirchhoff’s current law and is specified as follows:
  9 $${I}_{\text{out }}={I}_{PC}-{I}_{rsD}\left({e}^{\frac{Q\left({V}_{l}+{I}_{\text{out }}{R}_{\text{se }}\right)}{(NKT)}}-1\right)-\frac{\left({V}_{l}+{I}_{\text{out }}{R}_{\text{se }}\right)}{\left({R}_{SH}\right)}$$

According to Eq. (9), ${V}_{l}$ represents the resultant voltage, $K$ symbolizes the Boltzmann constant, $Q$ symbolizes the electric charge of an electron, and $T$ represents the temperature measured in Kelvin. Hence, it is necessary to ascertain the values of the five unidentified parameters, namely ${I}_{rsD}$, ${I}_{PC}$, ${R}_{se}$, ${R}_{SH}$, and $N$, for the single diode model. Optimization approaches can be employed to precisely estimate these parameters in order to appropriately depict the solar cell’s efficiency. Accurate and strong parameter estimation is crucial for obtaining dependable outcomes.

**Fig. 2:**
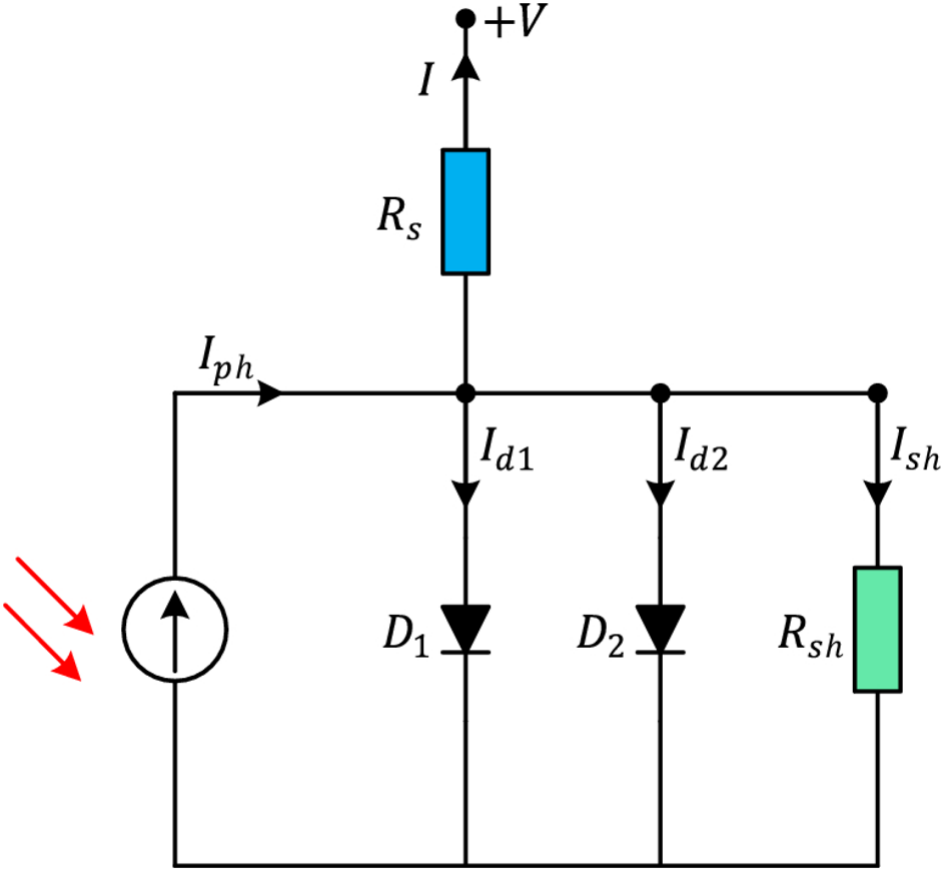
Circuit of Double diode model.

- The single-diode model has been broadly utilized to analyze the solar cells’ static characteristics because of its accuracy and simplicity. Nevertheless, this model is limited due to its assumption of a constant diode ideality factor throughout the whole range of output voltage. The diode ideality factor changes in response to the voltage applied across the device. In addition, the single-diode model fails to account for the effect of current recombination losses in the depletion zone. To tackle these concerns and improve the authenticity of the model, a *double-diode* model can be employed. Figure 2 demonstrates the inclusion of an extra recombination diode that runs parallel to the original diffusion diode in this model. The double-diode model’s output current is determined by the following equation:
  10 $$\begin{array}{l}{I}_{\text{out }}={I}_{PC}-{I}_{rsD1}\left({e}^{\frac{Q\left({V}_{l}+{I}_{\text{out }}{R}_{se}\right)}{(N1KT)}}-1\right)-{I}_{rsD2}\left({e}^{\frac{Q\left({V}_{l}+{I}_{\text{out }}{R}_{se}\right)}{(N2KTT)}}-1\right)\\ -\frac{\left({V}_{t}+{I}_{\text{out }}{R}_{\text{se }}\right)}{\left({R}_{SH}\right)}\end{array}$$

In this case, the diffusion current is denoted by ${I}_{rsD1}$ while the saturation current is represented by ${I}_{rsD2}$. Moreover, ${N}_{1}$ and ${N}_{2}$ represent the ideality factors for the diffusion and saturation currents, respectively. According to Eq. (10), the seven parameters that need to be estimated for the double-diode model are, ${N}_{1}$, ${I}_{rsD1}$, ${I}_{PC}$, ${R}_{SH}$, ${I}_{rsD2}$, ${R}_{se}$ and ${N}_{2}$. These parameters are crucial for accurately modeling the double-diode behavior.

**Fig. 3:**
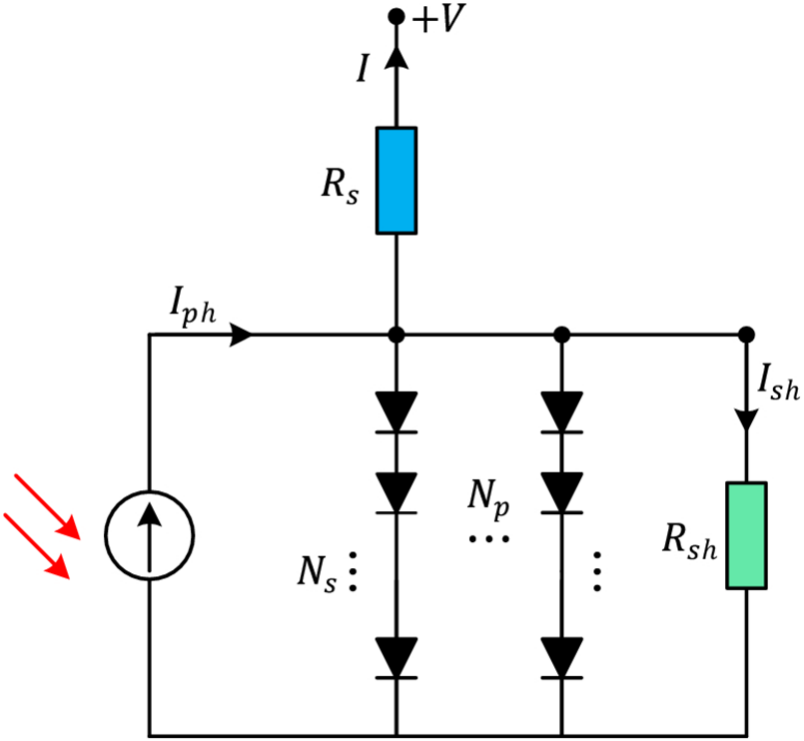
Circuit of PV module model.

- Finally, the Fig. 3 displays the layout of the PV module model. In this case, many solar cells are configured in parallel or series organization. Equations (11) and (12) can be used to describe the output current of PV modules based on both the double-diode and single-diode models, respectively.
  11 $$\begin{array}{l}{I}_{\text{out }}\\ ={C}_{p}{I}_{PC}-{C}_{p}{I}_{rsD1}\left({e}^{\frac{Q\left({V}_{l}+{C}_{s}{I}_{\text{out }}{R}_{se}/{C}_{p}\right)}{\left(N1KT{C}_{s}\right)}}-1\right)\\ -{C}_{p}{I}_{rsD2}\left({e}^{\frac{Q\left({V}_{l}+{C}_{s}{I}_{\text{out }}{R}_{se}/{C}_{p}\right)}{\left(N2KT{C}_{s}\right)}}-1\right)-\frac{\left({V}_{l}+{I}_{\text{out }}{R}_{se}{C}_{s}/{C}_{p}\right)}{\left({R}_{sH}{C}_{s}/{C}_{p}\right)}\end{array}$$
  12 $${I}_{\text{out }}={C}_{p}{I}_{PC}-{C}_{p}{I}_{rsD}\left({e}^{\frac{Q\left({V}_{l}+{C}_{s}{I}_{\text{out }}{R}_{se}/{C}_{p}\right)}{\left(NKT{C}_{s}\right)}}-1\right)-\frac{\left({V}_{l}+{I}_{\text{out }}{R}_{se}{C}_{s}/{C}_{p}\right)}{\left({R}_{sH}{C}_{s}/{C}_{p}\right)}$$

where the number of parallelly configured solar cells are denoted by ${C}_{p}$, while the number of serially configured solar cells are denoted by ${C}_{s}$. Therefore, the five parameters that must be estimated for the single-diode model-based PV module are $N$, ${I}_{rsD}$, ${I}_{PC}$, ${R}_{se}$ and ${R}_{SH}$ while the seven parameters required to be estimated for the double-diode model-based PV module are ${I}_{rsD1}$, ${I}_{PC}$, ${R}_{SH}$, ${I}_{rsD2}$, ${N}_{1}$, ${R}_{se}$, and ${N}_{2}$.

## Proposed methodology: EBMLO-DBO algorithm for global complex problem and parameter estimation of solar photovoltaic model

Among global search algorithms, the DBO algorithm stands out due to its major features: simplicity, minimum number of parameters and fast calculation power. In certain cases, its capacity to converge rapidly after a limited number of iterations allows it to find the global optimum. Diversity declines as iterations go on because agents in the population try to gravitate toward the best location. It might thus be vulnerable to local optima, which would prevent it from reaching optimal solutions. Reduced computational precision and sluggish convergence are the results of this contributing factor. Early on, good performance was guaranteed by the founding population’s equitable distribution and high quality. As a result, the Bernoulli map (BM) is utilized to provide diverse solutions and enhance the exploration phase of DBO. Furthermore, to improve the EBMLO-DBO’s exploitation potential and expand the search space, the Morlet Wavelet mutation (MWM) strategy is utilized. As a result, it improves convergence rate and computational accuracy. In addition, at the end of each EBMLO-DBO iteration, the Local Escape Operator (LEO) strategy is applied to improve the quality of solutions and establish a more optimal trade-off between investigating novel possibilities and utilizing already-existing solutions in the domain-based optimization process. Finally, the elite leadership strategy (ELS) is proposed to enhance the population depending on more elite with lower fitness values agents. The details of the proposed strategies are discussed in the following subsections.

### System model

This section explains the four main strategies applied to DBO algorithm in detail. Then, the working principle of the EBMLO-DBO algorithm is outlined to give a general overview for the proposed algorithm in its inherent working principle. Finally, the computational complexity of the proposed work is provided in terms of the time and space complexity.

#### Bernoulli map-based population initialization (BM)

MAs rely on iteratively refining candidate solutions, so their initial values are crucial for their performance in terms of convergence and exploration^78^. Commonly, the initialization phase of these algorithms involves generating random values within a specified range, often using a Gaussian distribution. This approach, however, depends significantly on randomness, which can hinder deeper insight and progress. Alternatively, chaotic maps can produce chaotic sequences—random-like sequences generated by simple deterministic systems. These maps exhibit properties such as non-linearity, sensitivity to initial conditions, randomness, ergodicity, fractional stability, chaotic attractors, local instability, long-term unpredictability, and global stability. In optimization, chaotic maps are often preferred over pseudo-random numbers for generating values between 0 and 1. Studies have demonstrated that employing chaotic sequences for tasks such as population initialization, selection, crossover, and mutation can enhance algorithm performance and frequently results in better convergence than using random numbers^79^. An example of a chaotic system is the Bernoulli map, a segmented chaotic system defined as follows:

13 $$z_{n + 1} = \left\{ {\begin{array}{*{20}l} {\frac{{z_{n} }}{1 - \alpha }} \hfill & {0 \le z_{n} \le 1 - \alpha } \hfill \\ {\frac{{z_{n} - \left( {1 - \alpha } \right)}}{\alpha }} \hfill & {1 - \alpha \le z_{n} \le 1} \hfill \\ \end{array} } \right.$$

where α, the mapping parameter, is between 0 and 1 which is usually set to 0.29^80^. One unique instance where these maps exist is:

14 $${z}_{n+1}=2\times {z}_{n}mod1$$

Therefore, the novel population initialization approach can be modeled as:

15 $${X}_{i}=Lb+{z}_{n+1}\times (Lb-Ub)$$

Therefore, new population initialization strategy using Bernoulli mechanism to enhance the diversity of the initial population. This approach leverages the chaotic properties of Bernoulli maps to ensure a more varied set of candidate solutions from the start, which significantly improves the exploration capabilities of the DBO algorithm. By increasing the diversity at the initialization phase, this strategy helps prevent premature convergence and enhances the algorithm’s overall performance.

#### Elite leadership strategy (ELS)

Upon each iteration’s completion, the elite leadership strategy^81^ divides the current population $X(t)$ into two subsets: the elite group $Y1(t)$ and the non-elite group $Y2(t)$. This division ensures that $Y1(t)$ and $Y2(t)$ are distinct and together comprise $X(t)$ where $Y1\left(t\right)\cup Y2\left(t\right)=X(t)$ and $Y1\left(t\right)\cap Y2\left(t\right)=\varnothing$. The size of the elite group is denoted as $\Gamma$. Three random members ${X}_{r1}$, ${X}_{r2}$, and ${X}_{r4}$ are picked from the elite group $Y1(t)$, while ${X}_{r3}$ and ${X}_{r5}$ are selected from the non-elite one $Y2(t)$. Therefore, the elite leadership strategy is formulated as:

16 $$\begin{array}{c}Xnew1 =\left\{\begin{array}{c}{X}_{r1}+F\cdot \left({X}_{r2}-{X}_{r3}\right)+F\cdot \left({X}_{r4}-{X}_{r5}\right), \text{ if rand }\le \tau \\ {X}_{best}+F\cdot \left({X}_{r2}-{X}_{r3}\right)+F\cdot \left({X}_{r4}-{X}_{r5}\right), \text{ otherwise}\end{array}\right.\end{array}$$

According to Eq. (16), ${X}_{best}$ is the top individual of generation $t$, $F$ represents the scaling factor, and rand is a random value between 0 and 1. The selection probability $\tau$ is calculated as:

17 $$\tau =\frac{1}{1+{e}^{1-(T/t{)}^{2}}}.$$

The selection in Eq. (16a) utilizes a process where individuals from $Y1$ are picked at random to provide guidance to the population. This selection process is referred to as elite random leadership. On the other hand, Eq. (16b), which utilizes the most optimal individual to generate new individuals, is referred to as best leadership. If the mutation strategy gives higher importance to global exploration, the algorithm may move towards the global best solution, but it will need a greater number of iterations. On the other hand, focusing on exploiting local resources can decrease the variety of search results, which in turn hampers the ability to explore new possibilities. As stated in Eq. (17), at the start of the iteration, the selection probability for elite random probability is almost 1, whereas the selection probability for optimal leadership is nearly 0. As the iterations progress, the algorithm gradually approaches the optimal solution by raising the probability of selecting the best leadership and decreasing the probability of selecting random leadership from the elite group. During this process of mutation, $Y1$ offers a favourable path for speeding up convergence, while $Y2$ aids in adjusting the search orientation, so improving population variety to avoid premature convergence. Finally, if the new position $Xnew1$ improves the objective function value of the current population solutions, as detailed in Eq. (18), it is accepted for replacement of the current solution as follows:

18 $${X}_{i}\left(t+1\right)=\left\{\begin{array}{c}Xnew1, f\left(Xnew1\right)<f\left({X}_{i}\right)\\ {X}_{i}\left(t\right), otherwise\end{array}\right.$$

The ELS strategy plays a critical role in enhancing the performance of the DBO algorithm. The population is split into elite and non-elite groups, and scope for mutation is induced by choosing certain individuals which guarantees that both exploration and exploitation strategies are maintained. The early usage of elite random leadership allows exploring diversity and global search during the initial phases while over time starting to introduce the better leadership allows to better progress the search towards better solutions. This dynamic adjustment helps reduce or minimizes the possibility of early convergence as well as enhances the performance of the solutions when solving local optima. As a result, the ELS strategy considerably raises the efficiency and effectiveness of the EBMLO-DBO algorithm at solving the problem of high-quality solutions.

#### Morlet wavelet mutation strategy (MWM)

Swarm intelligence optimization algorithms rely on jumping out of the local optimum when solving problems with dense distributions of extreme points. The primary driver of biological evolution is mutation^10^. In order to increase the stability of the solution, wavelet mutation (MWM) can dynamically modify the mutation space using the wavelet function’s translation and scaling capabilities^82^. This study integrates the DBO algorithm with wavelet mutation. In order to achieve the fine-tuning impact of the mutation operation, improve the ability to jump out of the local optimum, and increase the algorithm’s calculation accuracy, the number of iterations in the wavelet function’s scaling parameters is used to confine the mutation space. Mutation is only carried out during the exploratory phase of the algorithm since its goal is to break out of the local optimum.

Let $p$ be the mutation probability on the interval [0,1], and $s$ be a random number. Mutation probability indicates that when $s\ge p$, dung beetles experience Morlet wavelet mutation, specifically:

when $$i=1$$,

19 $$\begin{array}{c}{X}_{i}^{d}(t+1)=\left\{\begin{array}{c}{X}_{\text{rand }}^{d}+s\cdot \left({X}_{\text{rand }}^{d}(t)-{X}_{i}^{d}(t)\right)+\\ +\beta \cdot \left({X}_{\text{rand }}^{d}(t)-{X}_{i}^{d}(t)\right)+\sigma \cdot \left(U{b}^{d}-{X}_{i}^{d}(t)\right), \text{ rand }>0.5\\ {X}_{\text{rand }}^{d}+s\cdot \left({X}_{\text{rand }}^{d}(t)-{X}_{i}^{d}(t)\right)\\ +\beta \cdot \left({X}_{\text{rand }}^{d}(t)-{X}_{i}^{d}(t)\right)+\sigma \cdot \left({X}_{i}^{d}(t)-L{b}^{d}\right), \text{ rand }\le 0.5\end{array}\right.\end{array}$$

when $$i=2,\dots ,N$$,

20 $$\begin{array}{cc}& {X}_{i}^{d}(t+1) =\left\{\begin{array}{c}{X}_{\text{rand }}^{d}+s\cdot \left({X}_{i-1}^{d}(t)-{X}_{i}^{d}(t)\right)\\ +\beta \cdot \left({X}_{\text{rand }}^{d}(t)-{X}_{i}^{d}(t)\right)+\sigma \cdot \left(U{b}^{d}-{X}_{i}^{d}(t)\right), \text{ rand }>0.5\\ {X}_{\text{rand }}^{d}+s\cdot \left({X}_{i-1}^{d}(t)-{X}_{i}^{d}(t)\right)\\ +\beta \cdot \left({X}_{\text{rand }}^{d}(t)-{X}_{i}^{d}(t)\right)+\sigma \cdot \left({X}_{i}^{d}(t)-L{b}^{d}\right), \text{ rand }\le 0.5\end{array}\right.\end{array}$$

The point, ${X}_{rand}^{d}$, is randomly generated within the search space, $U{b}^{d}$ and $L{b}^{d}$. represents the upper and lower boundaries of the ${d}^{th}$ dimension, respectively. The wavelet mutation coefficient, $\sigma$, is defined as $\sigma =1/\sqrt{a}\psi (\varphi /a)$, $\psi (x)$ represents the Morlet wavelet function, which may be expressed as $\psi (x)={e}^{-{x}^{2}/2}\cdot cos(5x)$. The scaling parameter $a$ is incremented as the number of iterations increases in order to prevent the algorithm from overlooking the global best solution as a result of excessive mutation in the later stages of iteration which is defined as follows:

21 $$a=\lambda \cdot {\left(\frac{1}{\lambda }\right)}^{\left(1-\frac{t}{T}\right)},$$

where $\lambda$ is a given constant selected randomly from the range $[800, 1200]$.

Incorporation of the MWM strategy in EBMLO-DBO can be expected to further improve the performance, as it tends to provide better convergence speed and avoidance of local optima. This strategy will change the mutation space dynamically by translation and scaling properties in the wavelet function and solve solutions in much more stable and precise ways. Wavelet mutation can provide the ability for the algorithm to fine-tune its search capabilities, especially during the exploratory phase, hence increasing the possibility of the algorithm to get out of the local optima. The progressive adjustment of the scaling parameter $a$ helps maintain an effective balance between exploration and exploitation and ensures the algorithm will not miss the global best solution due to excessive mutation. This, therefore, assures such a procedure that it accelerates the convergence of the EBMLO-DBO algorithm, its robustness, and its accuracy toward the determination of an optimal solution.

#### Local escaping operator (LEO)

The Local Escaping Operator (LEO) is an additional local search approach^83^. Its main contribution is that it improves the search capability of the DBO algorithm, helping to locate unexplored areas of the search space-especially in those difficult real-world applications. This will result in an improvement of the general quality of a solution. LEO improves the convergence behavior of the optimization algorithm by updating solution positions based on specific criteria, hence preventing it from becoming trapped in local optima. LEO utilizes range of important tactics to offer alternative solutions that demonstrate superior performance. These tactics involve using the most favorable position, labeled as ${X}_{best}$, together with two randomly generated solutions known as ${X}_{r1}$ and ${X}_{r2}$. In addition, LEO randomly chooses two solutions, $X{1}_{i}$ and $X{2}_{i}$. Finally, LEO develops a novel solution, ${X}_{z}$, which is generated randomly. The suggestion outlines a mathematical calculation that can be used to calculate the value of ${X}_{LEO}$ as follows:22

The given equations have several variables. The variable $f1$ follows a uniform distribution with a range from -1 to 1, including both endpoints. The variable $f2$ follows a normal distribution with a mean of 0 and a standard deviation of 1. In addition, there are three additional random variables: $u1$, $u2$, and $u3$. Also, $\rho 1$​ denotes the likelihood.

23 $${u}_{1}=\left\{\begin{array}{ll}2\times \text{ rand }& \text{ if }{\mu }_{1}<0.5\\ 1& \text{ otherwise}\end{array}\right.$$

24 $${u}_{2}=\left\{\begin{array}{ll}\text{ rand }& \text{ if }{\mu }_{1}<0.5\\ 1& \text{ otherwise}\end{array}\right.$$

25 $${u}_{3}=\left\{\begin{array}{ll}\text{ rand }& \text{ if }{\mu }_{1}<0.5\\ 1& \text{ otherwise}\end{array}\right.$$

where the variable $rand$ indicates a randomly generated number that is within the range of 0 and 1. In contrast, the variable $\mu$ represents a number that is also constrained inside the range of 0 to 1. The provided equations can be simplified as shown in Eqs. (26–28):

26 $${u}_{1}={Q}_{1}\times 2\times rand+\left(1-{Q}_{1}\right)$$

27 $${u}_{2}={Q}_{1}\times rand+\left(1-{Q}_{1}\right)$$

28 $${u}_{3}={Q}_{1}\times rand+\left(1-{Q}_{1}\right)$$

The binary parameter $Q1$ can only have the values of 0 or 1. The value is determined by a condition: if $Q1$ is less than 0.5, then $Q1$ is assigned a value of 1. Alternatively, it is given a numerical value of zero. In addition, to ensure a fair allocation of resources between exploration and exploitation in search processes, the variable $\rho 1$ is included. The variable is determined using Eqs. (29–31):

29 $${\rho }_{1}=2\times rand\times y-y$$

30 $$y=\left|sin\left(sin\left(\beta \times \frac{3\pi }{2}\right)+\frac{3\pi }{2}\right)\times \beta \right|$$

31 $$\beta =\left({\beta }_{max}-{\beta }_{min}\right)+{\beta }_{min}\times {\left(1-{\left(\frac{t}{T}\right)}^{3}\right)}^{2}$$

The values of ${\beta }_{min}$ and ${\beta }_{max}$ are assigned as 0.2 and 1.2, respectively. The variable $t$ denotes the current iteration, whereas $T$ is the maximum number of iterations. In order to maintain a proper equilibrium between exploration and exploitation, the parameter $\rho 1$ dynamically adapts itself according to the sine function $y$. The solution for ${X}_{z}$ in the given scheme can be computed by applying the method described in Eqs. (32) and (33):

32 $${X}_{z}=\left\{\begin{array}{ll}{X}_{\text{rand }}& \text{ if }{\mu }_{2}0.5\\ {X}_{p}& \text{ otherwise}\end{array}\right.$$

33 $${X}_{\text{rand }}={X}_{\text{min }}+rand(\text{0,1})\times \left({X}_{\text{max }}-{X}_{\text{min}}\right)$$

From Eq. (32), the solution ${X}_{rand}$ denotes a novel solution, whereas ${X}_{p}$ represents a solution that has been chosen randomly from a population. Moreover, $\mu$ represents a random variable that follows a uniform distribution with values ranging from 0 to 1. Equation (32) can be simplified in the following manner:

34 $${X}_{z}={Q}_{2}\times {X}_{p}+\left(1-{Q}_{2}\right)\times {X}_{\text{rand}}$$

where the parameter ${Q}_{2}$ is a binary variable that can only take the values of 0 or 1. The value is determined based on the condition of $\mu$ being less than 0.5 or not. The stochastic selection of parameter values, ${u}_{1}$, ${u}_{2}$, and ${u}_{3}$, enhances the heterogeneity of the population and mitigates the likelihood of suboptimal solutions.

### EBMLO-DBO for local optima avoidance

Local optima represent a fundamental challenge in optimization where algorithms become trapped in suboptimal solutions, mistakenly treating them as global optima due to insufficient exploration or premature convergence^84^. In the EBMLO-DBO, local optima situations are systematically identified and addressed through multiple complementary strategies designed to maintain population diversity and exploration capability throughout the optimization process. Local optima conditions typically manifest when population diversity decreases below critical thresholds, fitness improvement stagnates over consecutive iterations, and search agents converge prematurely to limited regions of the search space, effectively terminating the search process before discovering superior solutions.

Thus, EBMLO-DBO employs four synergistic mechanisms to avoid local optima entrapment effectively. The Bernoulli map-based initialization generates chaotic sequences with inherent properties of non-linearity, sensitivity to initial conditions, and ergodicity, ensuring diverse starting positions that significantly reduce the probability of early convergence to local optima by providing better coverage of the search space. Morlet Wavelet mutation is strategically applied when stagnation is detected through the condition $s\ge p$ in Eqs. (19) and (20), where the wavelet function dynamically adjusts mutation space using the scaling parameter '$a$' that decreases with iterations according to Eq. (21), providing strong exploration capabilities in early optimization stages and fine-tuning adjustments in later stages. The ELS strategy prevents premature convergence by maintaining both elite random leadership and best leadership mechanisms, where the selection probability $\tau$ defined in Eq. (17) starts at approximately 1 and gradually decreases, ensuring continued exploration while progressively focusing on promising regions. The LEO strategy, activated with probability $pr$ through Eqs. (22–34), generates alternative solutions using the best position $Xbest$ and randomly selected solutions, effectively enabling the algorithm to “jump out” of local optima through strategic solution perturbations. The synergistic effect of these mechanisms ensures that even when individual agents become trapped in local optima, the overall population maintains sufficient diversity and exploration capability to escape and continue searching for global optima, with statistical analysis confirming that EBMLO-DBO successfully avoids local optima in most of test functions compared to the original DBO algorithm.

### Architecture and working

To balance the trade-offs between exploration and exploitation in solving complex optimization problems with enhanced efficiency, the proposed EBMLO-DBO is equipped with four advanced strategies. The step-by-step procedure of the EBMLO-DBO algorithm is elaborated in detail in this section. The algorithm progresses through a series of iterative updates, applying different strategies to various subsets of the population, with the ultimate goal of finding the optimal solution.

- **Step 1 Initialization:** The algorithm initializes the population according to Bernoulli maps presented in Eq. 15; this generates a highly diversified set of initial solutions since it gives a good starting point to efficiently explore the search space.
- **Step 2 Fitness evaluation:** This step involves the calculation and sorting of fitness values in each iteration. Ranking provides important information about the best solutions and about the splitting of the population and its update.
- **Step 3 Population division:** This is followed by the division of the population, based on the ranking, into two subgroups, $Y1(t)$ and $Y2(t)$, on which different strategies can be applied for an effective optimization of both exploration and exploitation.
- **Step 4 Rolling Dung Beetles Update:** For each rolling dung beetle, a random value $\alpha$ is generated. If $\alpha \le 0.9$, then the position of the rolling dung beetle is updated by Eq. (2). Otherwise, if the beetle hits the obstacle, its position will be updated by Eq. (3).
- **Step 5 Calculation of Convergence Factor:** The convergence factor $R$ will be computed using the relation $R=1-t/T$, where $t$ denotes the iteration at each run and $T$ signifies the maximum number of iterations. This factor is affecting the dynamics of the search during the runtime; thus, the balance between exploration and exploitation will be changing with time passing.
- **Step 6 Spawning Dung Beetles Update:** The position of spawning dung beetles is updated by using Eq. (4) and Eq. (5). Both these updates ensure that the spawning dung beetles contribute towards maintaining the diversity in population and enhance the process of searching.
- **Step 7 Foraging Dung Beetles Update:** If a random generated value is less than a predefined probability $p$, update the foraging dung beetle using Eq. (6) and Eq. (7); otherwise, apply Morlet Wavelet Mutation using Eq. (19) or Eq. (20) to introduce diversity for the avoidance of premature convergence.
- **Step 8 Stealing Dung Beetles Update:** The stealing dung beetles are updated using Eq. (8). This strategy focuses on exploiting the best areas of the search space, driven by the behavior of elite individuals.
- **Step 9 Elite Leadership and Greedy Selection:** Each solution undergoes an update using the elite leadership strategy as defined in Eq. (16). Afterward, a greedy selection process, guided by Eq. (18), compares new and old solutions to retain only the best candidates for the next iteration.
- **Step 10 LEO Operator Application and Boundary Check:** The Local Escape Operator (LEO) is applied to each solution using Eqs. (22–34), helping to escape local optima. Following this, all solutions are checked to ensure they remain within the predefined boundary limits.
- **Step 11 Update Optimal Solution:** The current best solution ${X}_{best}$​ is updated based on the latest evaluations. This ensures that the algorithm continuously tracks the most promising solution throughout the optimization process.
- **Step 12 Iteration and Completion:** The iteration counter $t$ is incremented, and the process repeats until the maximum number of iterations $T$ is reached. Upon completion, the algorithm returns the optimal solution ${X}_{best}$ along with its corresponding fitness value.

**Figure 4:**
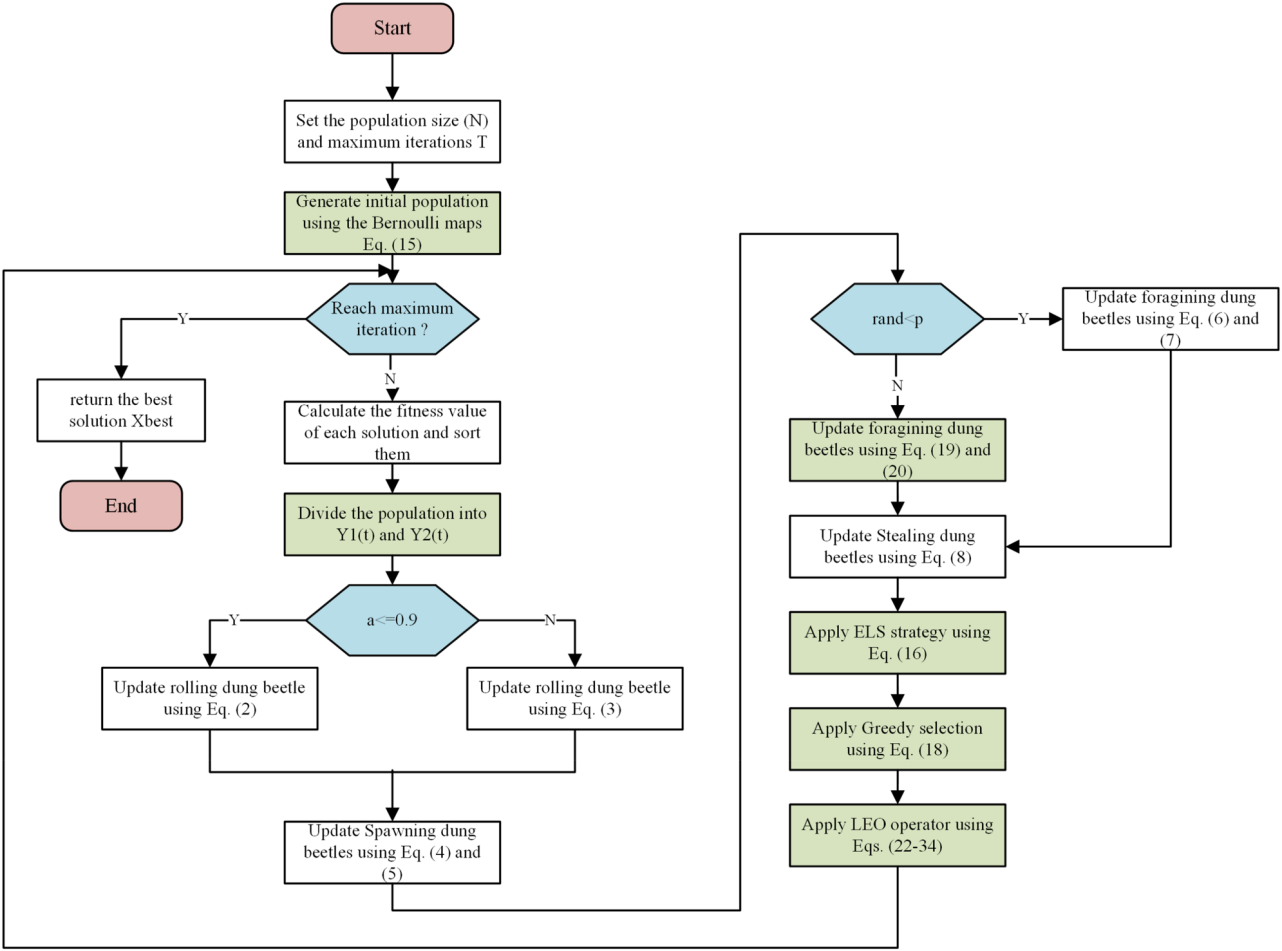
Proposed EBMLO-DBO algorithm.

The procedures and operators of the proposed EBMLO-DBO are depicted in Algorithm 1 and Fig. 4.

**Figure:**
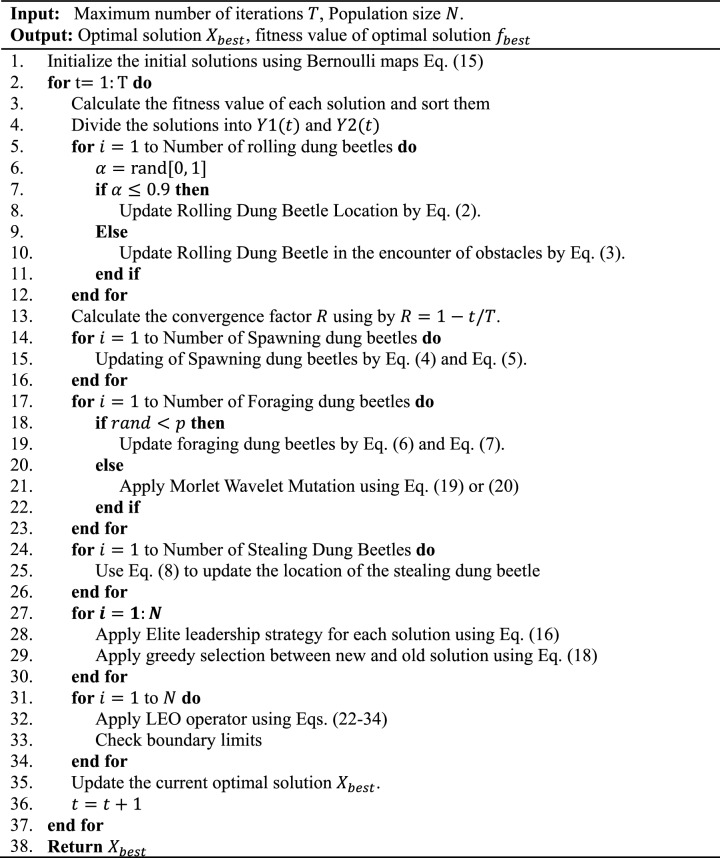
Algorithm 1: EBMLO-DBO algorithm

### Computational complexity

The computational complexity of an algorithm is an essential factor that affect its performance. The computational of an algorithm can depend on two parameters: the time complexity and the space complexity^85^. The time complexity assures and evaluate the theoretical performance of an algorithm in terms of its effectual operations while the space complexity is the required memory and storage for the algorithm while execution. The next subsections discuss the time and space complexity of EBMLO-DBO in details and compare it with the original DBO to assure the difference between them.

#### Time complexity

The variables dimension $D$, population size $N$, and number of iterations $T$ determine the algorithm’s temporal complexity. The original DBO is mainly depend on its internal operators beside the initialization phase. The main loop of the DBO algorithm include the four main phases of ball rolling, foraging, spawning and stealing. The main population is divided among these operators therefore, the main loop complexity includes the complexity of the four phases. The complexity of DBO can be described as follows:

$$O\left(DBO\right)=O\left(O(Initialization\right)+T O(operators)$$

$$=O\left(N\times D+T\times N\times D\right)$$

The inclusion of LEO, BM, MWM and ELS strategies are the extended operators found in EBMLO-DBO but not in DBO. In other words, every solution undergoes the BM which is used for the initialization require $O(N\times D)$ while the LEO is applied for every solution at each iteration, resulting in a complexity of $O(T\times N\times D)$. Since the MWM strategy is a component of the EBMLO-DBO update methodology, its complexity when paired with the operators of the DBO algorithm is $O(T\times N\times D)$. Since the ELS strategy is used at the conclusion of every iteration, its complexity is $O(T\times N\times D)$. In summary, EBMLO-DBO’s temporal complexity is:

$$O\left(EBMLO-DBO\right)=O\left(N\times D\right)+O\left(T\left(N\times D+N\times D+N\times D\right)\right)$$

$$=O(N\times D+T\times N\times D)$$

Therefore, when comparing DBO with the suggested EBMLO-DBO, the time complexity remains the same, but there are variations in performance.

#### Space complexity

The quantity of storage space required for an algorithm to execute is referred to as its space complexity. Thus, beginning from the initialization step, the space complexity of $O(N\times D)$ is the same for both DBO and the proposed EBMLO-DBO.

## Experimental results

This section highlights the experimentation results of the proposed EBMLO-DBO algorithm. First, we provide details about the experimental setup and parameter settings, which are crucial to replicate the study or take cognizance of the operating conditions in the algorithm evaluation. Then, the performance metrics used in the effectiveness evaluation of the algorithm are presented, completing the framework of comprehensive evaluation. Lastly, the section highlights, the test results from the experiments, presenting a comprehensive analysis in order to outline the strengths and weaknesses, along with the overall performance of the algorithm in question for the various test scenarios.

### Experimentation setup and parameter settings

This work compared the performance of the EBMLO-DBO algorithm against eight state-of-the-art algorithms that experience superior performance, together with eleven conventional and recent algorithms. In this work, a total of 41 benchmark functions were tested, including 12 from CEC’22^86^ and 29 from CEC’17^87^. This is supported by five modern algorithms: Crayfish Optimization Algorithm (COA)^20^, Levy Flight Distribution optimizer (LFD)^88^, Nutcracker Optimizer (NOA)^16^, Gradient-based Optimizer (GBO)^83^, original DBO, and six traditional algorithms: Particle Swarm Optimizer (PSO)^14^, Harris Hawk Algorithm (HHO)^89^, Arithmetic Optimization Algorithm (AOA)^90^, Whale Optimization Algorithm (WOA)^91^, Slime Mould Optimizer (SMA)^92^, and Artificial Rabbit Algorithm (ARO)^93^. Table 1 summarizes the conditions of these comparative schemes. In Table 1, all algorithms are performed under the same condition with the initial population size 30 and the same maximum iteration limit 1000. For each algorithm, results are generated by 30 runs for every test function in order to avoid random effects^94,95^. The mean best-of-run (AVG) and standard deviation (STD) of solutions are then recorded. All the experiments are conducted on an Intel(R) i7-10750H CPU computer running Microsoft Windows 10 with 32 GB of RAM. Besides, MATLAB R2020a is used as a development environment for coding to ensure computational power and dependability when conducting the experiments. The rank rows of the Tables show the ordering of the mean values. A rank of 1 means that, after 30 runs, the algorithm had the lowest mean of the solution; it therefore means higher search capacity.

**Table 1:** Comparative algorithms parameters’ values.

Algorithm	Setting values
PSO	$W=[0.4, 0.9], c1=c2=2$
HHO	${E}_{0}$ linear decrease from -1 to 1
AOA	$MOP limit=\text{0.2,1}$ $\alpha =5, \mu =0.499$
WOA	$Q=[-1, 1] and k=1$
SMA	$z=0.03$
ARO	$Depth weight=50, multiplier weight=0.2$
COA	${C}_{3}=3, \mu =25, \sigma =3$
NOA	${P}_{rp}=0.2, {P}_{a2}=0.4, \delta =0.05$
GBO	${\beta }_{min}=0.2, {\beta }_{max}=1.2$
LFD	$CSV=2, B=0.5, a1=1.5, a2=10, a3=0.00005$
DBO	$k=0.1, S=0.5 and b=0.3$

### Performance indicators

To accurately differentiate between algorithms and ensure a fair and credible comparison, this section introduces three key performance evaluation metrics—Mean and Standard Deviation—along with two statistical testing methods. These metrics and tests are utilized to compare the performance of the involved algorithms. For the ensuing, let $M$ be the number of independent runs of each algorithm, and for every run ${g}_{opt}$ will denote the best solution obtained. The specific definitions and calculation methods for these metrics are outlined below:

- **Average Value (AVG):** The mean value is a statistical measure that reflects the central tendency of the data^96^. It provides an overview of the typical performance of an algorithm over multiple runs. The calculation of the mean value is given by the following equation:
  35 $$\text{AVG }=\frac{1}{M}\times \left({g}_{opt}^{1}+{g}_{opt}^{2}+{g}_{opt}^{3}+\cdots +{g}_{opt}^{M}\right)$$
- **Standard Deviation (STD):** Standard deviation describes the dispersion of the individual observations with respect to the mean value. The higher is the STD, the larger is the dispersion, which in turn signifies that the algorithm produces more unstable results. A lower STD therefore means that a particular algorithm will produce more consistent and hence more stable results. The STD is calculated as follows:
  36 $$\text{STD }=\sqrt{\frac{{\left({g}_{opt}^{1}-AVG\right)}^{2}+{\left({g}_{opt}^{2}-AVG\right)}^{2}+\cdots +{\left({g}_{opt}^{M}-AVG\right)}^{2}}{M-1}}$$
- **Wilcoxon Rank Sum Test:** The Wilcoxon rank sum test is a non-parametric statistical test used to determine the significant median of differences between two independent samples. The test ranks all data from the two groups together to obtain the rank sums for each group and then compares these rank sums in order to show that the difference between the two groups is statistically significant. This approach is useful when considering two algorithms’ performances under different conditions.
- **Friedman Test:** The Friedman test is another non-parametric statistical test, but it compares three or more matched samples. In the case that there is more than one algorithm, the Friedman test will rank their performances from best to worst. Accordingly, each of these algorithms will be given a rank according to this test in terms of performance, and if there is no significant difference among the algorithms, their ranks should be evenly distributed. This test is supposed to indicate the performance of several algorithms relative to one another for various datasets or problem instances.

These performance metrics and statistical tests collectively provide a comprehensive framework for evaluating and comparing the effectiveness, stability, and reliability of different algorithms in a robust and statistically sound manner.

### Exploration- exploitation balance test

**Fig. 5:**
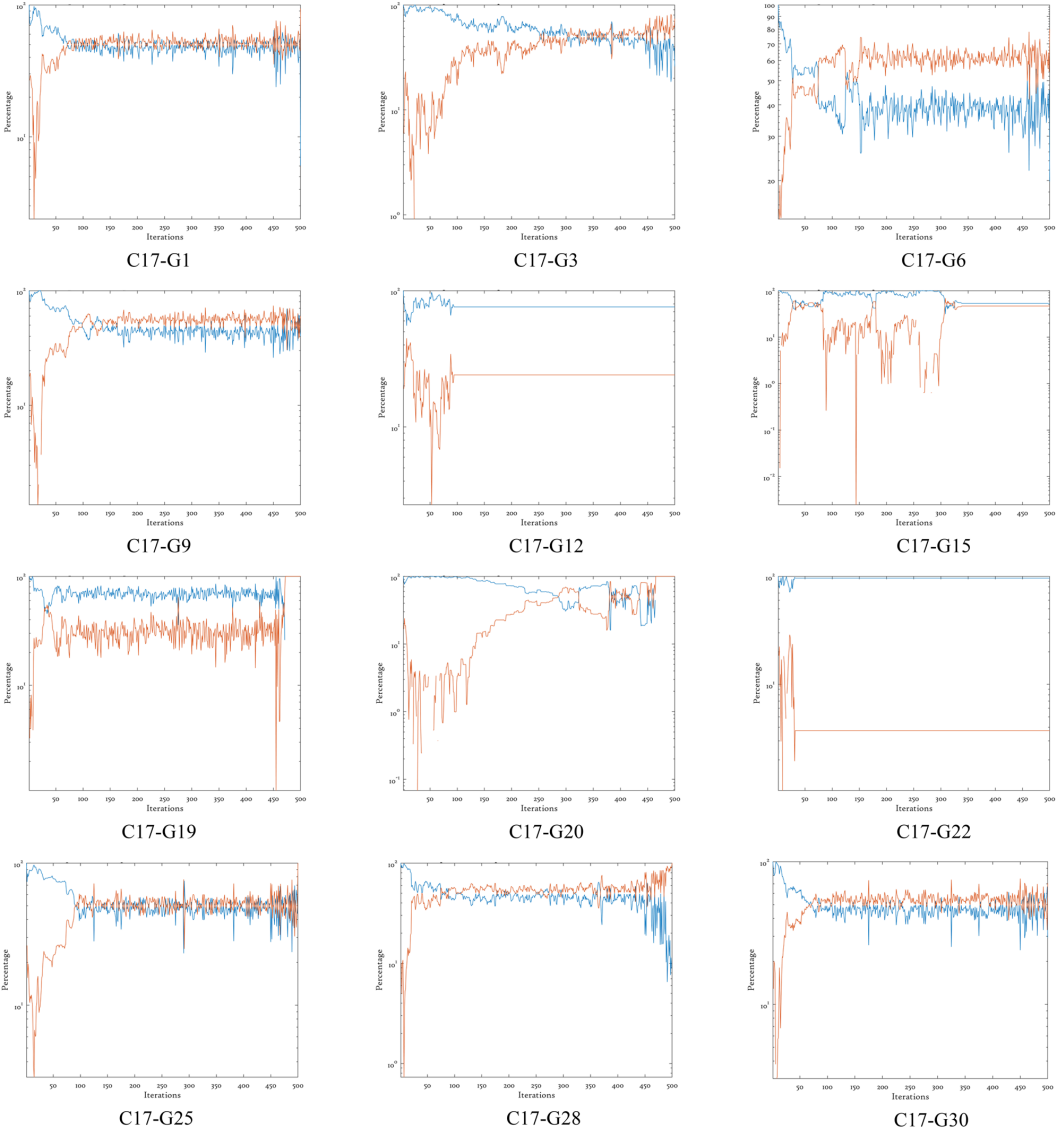
Exploration–exploitation analysis of different functions from CEC’17.

The exploration–exploitation percentage plots presented in Fig. 5 demonstrates the EBMLO-DBO algorithm’s superior ability to maintain an optimal dynamic balance between exploration and exploitation phases throughout the optimization process across diverse function landscapes from the CEC’17 benchmark suite. The blue line representing exploration percentages and the orange line representing exploitation percentages reveal that EBMLO-DBO consistently exhibits a well-coordinated transition from high exploration dominance in early iterations to increased exploitation in later stages, which is particularly evident in unimodal functions C17-G1 and C17-G3 where the algorithm maintains approximately 80–90% exploration during the initial 50 iterations before systematically transitioning to exploitation dominance with orange line values reaching 60–70% by the final iterations. The balanced approach is clearly visible in multimodal functions C17-G6 and C17-G9, where EBMLO-DBO demonstrates intelligent phase transitions with blue exploration percentages gradually decreasing from initial high values while orange exploitation percentages correspondingly increase, ensuring effective navigation through complex search landscapes with multiple local optima. The algorithm shows remarkable adaptability by maintaining sufficient exploration capability when encountering challenging regions while smoothly transitioning to exploitation mode once promising areas are identified.

The exploration–exploitation balance validation is further reinforced through EBMLO-DBO’s performance on hybrid functions C17-G12, C17-G15, C17-G19, and C17-G20, where the algorithm demonstrates exceptional balance control with blue exploration lines starting at high percentages and gradually decreasing while orange exploitation lines correspondingly increase, maintaining moderate exploration levels during mid-stage iterations to ensure comprehensive search coverage without premature convergence to suboptimal solutions. The composite functions C17-G22, C17-G25, C17-G28, and C17-G30 present the most challenging scenarios for balance maintenance, yet EBMLO-DBO consistently achieves stable exploration–exploitation transitions with smooth percentage curves, where the blue exploration line typically exhibits initial dominance of 80–90% that systematically decreases as the orange exploitation line increases to achieve dominance of 60–80% in later iterations. This dynamic balance mechanism is achieved through the coordinated interaction of the four enhancement strategies integrated within EBMLO-DBO, where the Bernoulli map initialization promotes diverse blue exploration percentages in early stages, the elite leadership strategy provides intelligent guidance for balanced transitions between blue and orange phases based on population quality, the MWM maintains necessary exploration capabilities during critical phases to escape local optima, and the LEO enhances orange exploitation effectiveness during convergence phases. The consistent and adaptive exploration–exploitation balance exhibited by EBMLO-DBO across all twelve function categories, as demonstrated by the smooth percentage transitions and appropriate phase dominance patterns in Fig. 5, directly contributes to its enhanced optimization performance and robust solution quality.

### Ablation study

This section presents a comprehensive ablation study designed to evaluate the individual contribution of each proposed enhancement strategy to the overall performance of the EBMLO-DBO algorithm. To systematically assess the impact of each component, six algorithmic variants were developed and tested: BDBO incorporates only the Bernoulli map-based initialization strategy, EDBO implements solely the ELS strategy, MDBO utilizes only the MWM approach, LDBO employs exclusively the LEO strategy, DBO represents the original baseline algorithm, and EBMLO-DBO integrates all proposed strategies collectively. The experimental evaluation was conducted using the CEC’22 benchmark suite comprising twelve distinct optimization functions.

The results presented in Table 2 demonstrate the substantial individual impact of each enhancement strategy when compared to the original DBO algorithm, with the EBMLO-DBO achieving the most significant improvements across all function categories. The LDBO variant, incorporating only the LEO strategy, showed remarkable performance gains over the original DBO, particularly in unimodal function C22-G1 where it achieved 2.04E + 04 compared to DBO’s 2.28E + 07, and in hybrid function C22-G6 with 39,527.408 versus DBO’s catastrophic 1.75E + 10, demonstrating LEO’s effectiveness in escaping local optima and enhancing exploitation capabilities. The MDBO variant, utilizing solely the MWM strategy, exhibited consistent improvements across most functions, achieving near-optimal performance in C22-G1 with 333.975 compared to DBO’s 2.28E + 07, and maintaining competitive results in multimodal and composite functions, indicating the wavelet mutation’s capacity to enhance exploration while preventing premature convergence. The EDBO variant with ELS strategy showed moderate but consistent improvements over the original DBO, particularly effective in hybrid functions C22-G5 and C22-G6 where it achieved 2,161.254 and 1.27E + 04 respectively compared to DBO’s significantly higher values of 2.64E + 04 and 1.75E + 10, while the BDBO variant with Bernoulli map initialization demonstrated mixed results, showing substantial improvements in some functions like C22-G1 but performing worse than DBO in certain composite functions, suggesting that chaotic initialization alone requires complementary strategies to achieve consistent optimization performance. The synergistic integration of all strategies in EBMLO-DBO consistently outperformed both the original DBO and all individual variants, achieving the best average rank of 1.83 and final Friedman rank of 2.16, while the original DBO ranked consistently last with an average rank of 5.75, confirming that each enhancement strategy addresses specific algorithmic limitations and their collective implementation provides the most robust optimization framework.

**Table 2:** Different variants of DBO using CEC’22.

Function	Metric	EBMLO-DBO	DBO	LDBO	EDBO	BDBO	MDBO
C22-G1	AVG	**308.803**	2.28E + 07	2.04E + 04	2.76E + 04	2.45E + 08	333.975
C22-G2	AVG	457.943	11542.300	511.769	514.565	11432.275	**449.652**
C22-G3	AVG	606.774	726.832	**601.200**	635.937	743.157	610.280
C22-G4	AVG	868.453	1170.626	**863.025**	925.434	1123.666	889.098
C22-G5	AVG	**1437.972**	2.64E + 04	1568.951	2161.254	1.51E + 04	1529.780
C22-G6	AVG	2704.023	1.75E + 10	39527.408	1.27E + 04	8.72E + 09	**1958.569**
C22-G7	AVG	2058.369	2788.722	**2054.511**	2153.314	2589.807	2064.490
C22-G8	AVG	2252.839	134931.075	**2224.936**	2370.708	18687.864	2230.203
C22-G9	AVG	**2480.781**	5155.567	2489.958	2500.358	4872.888	2480.781
C22-G10	AVG	2534.153	8440.528	2552.825	**2513.531**	8420.040	2572.765
C22-G11	AVG	2920.000	15973.765	2916.959	2966.474	16588.023	**2720.001**
C22-G12	AVG	**2965.008**	4829.578	2973.233	3024.357	4467.604	2982.819
Average rank		**1.83**	5.75	2.25	3.67	5.25	2.25
Final rank		**1**	6	2	4	5	3
Friedman Rank		**2.16**	4.26	2.57	3.92	5.45	2.64

### CEC’22 results analysis

**Fig. 6:**
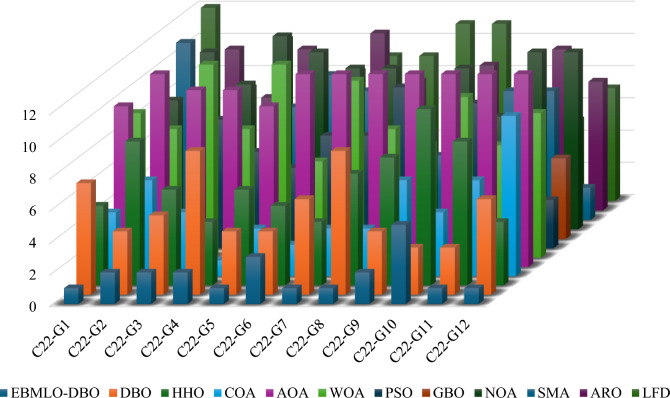
Average rank comparison between EBMLO-DBO and its rivals based on CEC’22, D = 20.

The CEC’22 test set comprises 12 distinct optimization test functions. In the experimental configuration, a dimension of 20 is employed. The comparisons have encompassed a range of algorithms, including DBO, WOA, HHO, LFD, PSO, GBO, NOA, AOA, COA, SMA, and ARO. CEC’22 has a greater degree of intricacy and challenge. The obtained results of applying EBMLO-DBO compared to its rivals using CEC’22 is shown in Table 3. EBMLO-DBO exhibits the best rank in the functions C22-G1, C22-G5, C22-G7, C22-G8, C22-G11 and G12, as evidenced by the statistical values presented in Table 3. Furthermore, the mean ranking of the solution outcomes for EBMLO-DBO and its corresponding algorithms, acquired from 30 separate runtimes of each function, is depicted in Fig. 6. Based on the data provided, it can be inferred that EBMLO-DBO consistently identifies superior solutions for these functions and maintains a high level of solution stability. EBMLO-DBO outperforms all other algorithms by achieving the highest rank in 50% of the functions and dominating them. On the other hand, EBMLO-DBO attains the second-highest position in 41.6% of the functions and the third-highest position in the remaining functions, surpassing all competitors. It is also analyzed that, the functions whose EBMLO-DBO is ranked the second, the gap between the second rank and the first is small. For example, for C22-G2, the PSO obtained the best rank in terms of the average fitness value but the stability of C22-G2 is the best indicating the superior performance of DBMLO-DBO at functions with the second rank. For the function C22-G3, the GBO was the best among all competitors this is due to the LEO operator which enhances the search ability of GBO at this function which indicates the powerful ability of the LEO operator as a new improvement proposed to EBMLO-DBO. Regarding the average rank, it is clear that the exceptional performance of EBMLO-DBO outperform all its rival while GBO is the second in terms of the average rank. The aforementioned investigations imply that EBMLO-DBO exhibits exceptional global search and optimization prowess in nearly all CEC’22 functions. It showcases the consistent capacity to consistently discover exceptional solutions and generate solutions that are of superior quality and stability.

**Table 3:** Comparative analysis between EBMLO-DBO and its rivals using CEC’22, D = 20.

F		EBMLO-DBO	DBO	HHO	COA	AOA	WOA	PSO	GBO	NOA	SMA	ARO	LFD
C22-G1	AVG	**301.65**	2384.85	1441.63	1026.37	3477.08	2947.99	467.47	2120.86	2927.99	4127.77	1551.16	4332.86
C22-G1	STD	**1.38**	9480.85	4839.38	4802.43	1161.74	1423.36	44.89	1225.83	9230.23	2429.82	4166.38	8001.90
C22-G1	RNK	1	7	5	4	10	9	2	3	8	11	6	12
C22-G2	AVG	454.80	490.85	617.29	526.27	2159.53	576.44	**436.40**	460.05	833.74	520.50	772.97	570.20
C22-G2	STD	**10.57**	74.93	124.45	46.00	654.54	64.67	26.57	13.16	121.00	102.94	104.10	95.51
C22-G2	RNK	2	4	9	6	12	8	1	3	11	5	10	7
C22-G3	AVG	615.18	630.15	633.53	626.55	664.25	665.93	648.76	**611.27**	649.49	621.71	645.93	654.28
C22-G3	STD	9.03	10.64	9.26	7.58	8.79	10.42	12.29	8.25	11.66	8.86	**4.74**	10.98
C22-G3	RNK	2	5	6	4	11	12	8	1	9	3	7	10
C22-G4	AVG	863.42	920.44	887.64	**849.53**	951.49	910.27	891.01	877.27	966.69	908.01	947.02	890.58
C22-G4	STD	16.89	35.04	20.20	14.58	20.53	32.01	19.82	**11.57**	19.19	32.73	11.68	26.82
C22-G4	RNK	2	9	4	1	11	8	6	3	12	7	10	5
C22-G5	AVG	**1408.87**	2126.38	2188.71	1966.60	2992.23	3459.59	2155.64	1759.07	3236.81	2859.30	2599.35	2609.75
C22-G5	STD	**337.59**	569.28	506.49	430.24	360.46	1210.14	809.62	406.44	1069.82	1168.63	509.75	734.23
C22-G5	RNK	1	4	6	3	10	12	5	2	11	9	7	8
C22-G6	AVG	7409.83	1281.41	4786.89	4418.48	8169.74	8060.06	2429.74	**3666.74**	6886.42	6338.33	1125.70	4652.90
C22-G6	STD	6984.63	2994.64	3100.92	4182.92	7343.12	1070.03	9704.27	**999.76**	5402.34	1286.00	1009.88	1760.87
C22-G6	RNK	3	4	5	2	12	6	7	1	10	8	11	9
C22-G7	AVG	**2065.15**	2121.14	2119.48	2107.80	2239.45	2232.14	2137.17	2073.61	2182.49	2120.57	2151.16	2159.75
C22-G7	STD	**22.86**	42.45	38.19	48.06	100.44	68.36	31.67	28.49	51.55	40.90	23.22	45.57
C22-G7	RNK	1	6	4	3	12	11	7	2	10	5	8	9
C22-G8	AVG	**2222.97**	2322.80	2296.54	2243.55	2533.33	2309.11	2360.31	2233.24	2295.53	2267.97	2280.31	2375.01
C22-G8	STD	**3.11**	78.26	79.70	51.39	201.74	78.25	127.65	22.46	63.39	54.64	26.05	123.18
C22-G8	RNK	1	9	7	3	12	8	10	2	6	4	5	11
C22-G9	AVG	2480.80	2499.83	2576.28	2509.93	3259.03	2564.27	**2467.72**	2480.81	2630.88	2506.91	2592.03	2666.02
C22-G9	STD	**0.03**	20.30	52.59	20.54	334.72	43.54	0.68	0.04	40.32	31.16	29.35	92.29
C22-G9	RNK	2	4	8	6	12	7	1	3	10	5	9	11
C22-G10	AVG	2966.22	2799.31	4809.55	2901.22	5781.02	4794.52	4284.40	2578.70	3351.42	4087.10	3024.05	**2524.17**
C22-G10	STD	564.07	773.16	1359.21	748.25	788.44	1141.27	1174.42	174.22	1388.81	983.52	1202.27	**84.16**
C22-G10	RNK	5	3	11	4	12	10	9	2	7	8	6	1
C22-G11	AVG	**2920.00**	3065.81	4112.37	3574.24	8414.46	3842.55	3099.51	2920.00	5016.85	3953.99	4755.92	3419.93
C22-G11	STD	**40.68**	203.75	455.74	377.67	772.99	1487.05	82.65	76.11	706.85	772.70	506.43	535.32
C22-G11	RNK	1	3	9	6	12	7	4	2	11	8	10	5
C22-G12	AVG	**2954.73**	3014.55	2975.79	3118.69	3803.62	3087.48	2967.22	2980.13	3257.88	2958.80	3068.05	3015.53
C22-G12	STD	**11.83**	49.11	19.20	79.32	218.87	114.95	121.22	28.25	82.75	13.99	32.07	156.73
C22-G12	RNK	1	6	4	10	12	9	3	5	11	2	8	7
Average rank		1.83	5.33	6.50	4.33	11.50	8.92	5.25	2.42	9.67	6.25	8.08	7.91
Final rank		**1**	5	7	3	12	10	4	2	11	6	9	8

### Comparison of EBMLO-DBO with advanced algorithms

To further evaluate the performance of EBMLO-DBO against high-performing algorithms, an experiment was conducted using eight advanced optimization techniques. These include five advanced algorithms: DAOA^97^, SCADE^98^, RLTLBO^99^, , and LWOA^100^ along with three top-performing algorithms in the CEC competitions: CMAES^101^, LSHADE-cnEpSin^102^, IMODE^103^, and AGSK^104^. The experiment involved 30 independent runs on twenty-nine CEC’17 benchmark functions with a dimensionality of 50, and the results are summarized in Table 4, highlighting the mean and standard deviation of the fitness values.

**Table 4:** Comparative analysis between EBMLO-DBO and its high-performing rivals using CEC’17.

F		EBMLO-DBO	DBO	CMAES	IMODE	AGSK	DAOA	SCADE	LSHADE_cnEpSin	RLTBLO	LWOA
C17-G1	AVG	**14,311.49**	4.73E + 09	1.37E + 10	6.05E + 05	1.06E + 07	2.57E + 11	2.68E + 11	2.65E + 09	5.22E + 09	8.89E + 10
C17-G1	STD	**9259.22**	1.10E + 10	2.41E + 10	4.32E + 05	1.63E + 07	2.73E + 10	2.57E + 10	2.58E + 09	2.52E + 09	1.05E + 10
C17-G3	AVG	**8870.45**	23,434.56	3867.07	2327.62	2184.12	9.60E + 07	5.11E + 11	1238.88	1264.88	1836.75
C17-G3	STD	**1426.11**	5094.31	4823.94	2640.71	3326.63	4.71E + 08	1.10E + 12	23,803.01	19,748.53	31,896.48
C17-G4	AVG	**517.08**	967.01	7680.90	557.92	612.15	1182.63	1218.11	935.51	935.06	2814.83
C17-G4	STD	56.85	376.79	1962.53	**40.07**	48.19	1921.12	2170.70	201.82	211.57	5797.22
C17-G5	AVG	799.05	992.46	**591.76**	746.74	916.39	1736.49	1717.66	844.10	728.05	1096.34
C17-G5	STD	**18.88**	95.80	144.22	50.88	33.07	74.39	74.59	49.09	71.15	48.29
C17-G6	AVG	**600.98**	664.16	637.04	642.53	619.25	755.92	756.25	643.23	616.20	682.74
C17-G6	STD	**0.28**	13.31	33.32	8.46	4.77	9.07	10.69	9.75	4.67	5.28
C17-G7	AVG	**899.92**	1473.01	1260.29	1030.22	1211.28	6147.38	6096.32	1385.82	1072.56	1882.78
C17-G7	STD	122.11	157.74	124.73	**26.45**	50.36	402.59	557.75	161.67	82.68	96.90
C17-G8	AVG	1081.37	1293.96	**1014.83**	1050.57	1215.54	1969.57	2075.92	1166.65	1057.18	1400.16
C17-G8	STD	43.74	82.59	244.80	**24.25**	37.17	99.69	75.70	62.62	81.25	43.62
C17-G9	AVG	1253.10	2159.09	**2893.30**	8888.37	5508.81	1062.16	1070.29	1947.00	8965.21	2549.66
C17-G9	STD	4986.78	9013.93	5266.97	**2247.02**	2758.89	1333.94	1129.65	7172.96	4743.20	3486.59
C17-G10	AVG	**7481.17**	1066.01	1491.08	8712.64	1216.16	1708.93	1846.53	1157.21	8798.23	1395.17
C17-G10	STD	1572.97	1967.13	412.39	579.36	**363.74**	483.42	756.07	1787.41	2743.80	815.85
C17-G11	AVG	**1397.58**	2619.20	7144.65	1400.89	1558.94	9017.99	8166.81	1514.56	3906.10	1927.02
C17-G11	STD	**76.09**	1314.14	1508.71	86.14	104.97	3029.03	2119.97	155.33	1225.80	4297.93
C17-G12	AVG	**3.66E + 06**	6.75E + 08	2.07E + 10	7.02E + 06	9.15E + 06	1.45E + 11	1.46E + 11	2.72E + 07	8.54E + 08	4.73E + 10
C17-G12	STD	**2.43E + 06**	7.47E + 08	5.34E + 09	3.86E + 06	5.49E + 06	2.38E + 10	3.12E + 10	3.02E + 07	1.39E + 09	1.11E + 10
C17-G13	AVG	54,765.99	5.13E + 07	1.07E + 10	1869.40	**1237.76**	9.18E + 10	9.00E + 10	31,435.71	1.22E + 08	2.36E + 10
C17-G13	STD	3186.91	6.95E + 07	2.90E + 09	2373.30	**1450.71**	1.87E + 10	2.96E + 10	4660.48	1.49E + 08	9.25E + 09
C17-G14	AVG	2580.94	3.11E + 06	2.13E + 07	3156.79	6217.66	3.91E + 08	5.19E + 08	**6150.35**	1.29E + 06	2.34E + 07
C17-G14	STD	**5092.37**	3.73E + 06	1.30E + 07	4068.38	1642.30	1.62E + 08	2.53E + 08	5795.46	1.37E + 06	2.52E + 07
C17-G15	AVG	**1310.94**	2.15E + 07	1.82E + 09	1328.79	2019.09	2.86E + 10	3.85E + 10	1556.25	1.10E + 07	3.94E + 09
C17-G15	STD	7629.29	7.20E + 07	7.82E + 08	1099.53	1258.66	9.26E + 09	9.04E + 09	**5517.40**	1.69E + 07	2.29E + 09
C17-G16	AVG	**3231.84**	4870.55	6785.58	3636.75	4103.92	1547.57	1690.99	3269.43	3735.16	6624.68
C17-G16	STD	524.61	539.93	435.46	261.32	**196.75**	3479.41	3720.65	417.83	521.91	687.00
C17-G17	AVG	3305.55	4188.26	**2850.38**	3021.02	3389.74	1408.86	2066.53	3183.48	2933.99	4311.43
C17-G17	STD	332.49	475.88	357.98	**143.31**	160.85	1915.20	2006.32	349.13	365.77	701.51
C17-G18	AVG	**7776.28**	8.16E + 06	1.05E + 08	2.33E + 06	1.24E + 06	6.47E + 08	1.56E + 09	1.33E + 06	5.25E + 06	3.98E + 07
C17-G18	STD	**4267.93**	9.49E + 06	5.63E + 07	1.68E + 06	7.78E + 05	2.47E + 08	7.40E + 08	8.64E + 05	4.80E + 06	2.63E + 07
C17-G19	AVG	2703.48	5.69E + 06	1.08E + 09	2.17E + 04	**1.14E + 04**	1.42E + 10	1.51E + 10	1.94E + 04	1.75E + 06	1.35E + 09
C17-G19	STD	1441.65	5.57E + 06	7.36E + 08	**5.94E + 03**	8.23E + 03	3.95E + 09	4.37E + 09	1.07E + 04	1.67E + 06	7.25E + 08
C17-G20	AVG	3155.05	3724.18	3770.03	3179.19	3454.81	5326.22	5771.62	3127.94	**3026.26**	3562.72
C17-G20	STD	369.33	295.64	259.76	216.60	**180.68**	220.49	341.67	231.17	356.99	314.03
C17-G21	AVG	**2511.78**	2846.25	2624.22	2543.19	2696.08	3576.64	3586.64	2570.49	2583.42	2975.29
C17-G21	STD	53.64	87.50	265.52	**26.37**	31.05	92.51	111.34	57.84	47.54	65.34
C17-G22	AVG	9968.14	1251.17	1649.78	8784.28	1360.63	1861.63	1999.41	**5614.02**	9974.86	1542.18
C17-G22	STD	2650.74	2187.30	**576.73**	2102.05	1803.22	621.97	788.78	3227.61	2351.02	915.49
C17-G23	AVG	**2983.49**	3509.43	3443.86	3011.27	3136.72	5369.55	5288.18	3120.22	3250.84	4002.30
C17-G23	STD	134.87	125.75	41.47	**24.14**	47.93	434.36	387.72	89.56	36.80	141.94
C17-G24	AVG	**3180.85**	3643.50	3521.97	3223.56	3282.49	6088.95	6083.19	3343.95	3423.47	4369.04
C17-G24	STD	135.49	132.39	38.43	**26.14**	49.22	524.73	479.51	88.63	107.24	302.58
C17-G25	AVG	**3066.35**	3985.12	3994.77	3107.01	3107.74	6356.42	6523.30	3410.29	3563.59	1357.74
C17-G25	STD	**31.46**	1943.41	1485.28	32.04	36.80	9647.88	1045.88	155.26	300.45	1604.26
C17-G26	AVG	**6547.37**	1038.31	1137.13	7523.39	7878.05	3395.51	3462.63	1059.68	6719.29	1602.28
C17-G26	STD	**262.58**	1400.09	486.78	2734.56	972.22	4959.49	5334.13	2200.85	757.99	876.51
C17-G27	AVG	3649.44	3917.74	3866.87	3546.68	**3504.30**	9013.99	9282.85	3691.17	3611.58	5588.63
C17-G27	STD	165.41	246.59	88.87	**51.63**	76.74	1219.60	1163.37	108.97	107.06	507.45
C17-G28	AVG	**3339.33**	6103.88	9714.15	3407.11	3404.15	2619.08	2517.11	3848.21	4159.27	1102.91
C17-G28	STD	37.77	2477.54	373.98	**29.62**	48.78	4049.05	3654.55	186.33	338.86	1086.70
C17-G29	AVG	**4141.04**	6271.43	1218.78	5142.31	4938.62	2284.31	4507.94	5164.77	4611.62	1560.33
C17-G29	STD	**223.57**	921.49	2862.13	415.37	239.80	2143.10	4263.54	432.96	321.01	6787.99
C17-G30	AVG	3.32E + 06	4.42E + 07	1.88E + 09	5.05E + 06	3.49E + 06	1.97E + 10	2.38E + 10	**1.43E + 06**	1.21E + 08	2.53E + 09
C17-G30	STD	1.70E + 06	4.81E + 07	6.47E + 08	1.10E + 06	1.56E + 06	5.27E + 09	6.49E + 09	**6.99E + 05**	4.23E + 07	1.76E + 09
Friedman rank		2.70	6.49	6.11	2.80	3.16	8.79	9.37	**4.14**	4.44	7.00

The investigation reveals that the EBMLO-DBO algorithm achieves the lowest average fitness value in 17 out of 29 functions, outperforming all other algorithms in the study. EBMLO-DBO also has the highest number of superior functions, demonstrating exceptional performance, particularly in unimodal functions CEC17-G1 and CEC17-G3, where it shows superior standard deviation and mean fitness values. For function CEC17-G4, while IMODE achieves the best standard deviation values, EBMLO-DBO secures the best average fitness values.

The original DBO method, in contrast, does not excel in any of the benchmark functions. The remarkable performance of EBMLO-DBO can be attributed to several proposed improvements. These include the MWM, which enhances exploration by introducing diverse solutions; the LEO, which helps the algorithm avoid local optima; the Bernoulli operator for initializing the population, which ensures diversity from the start; and the ELS strategy, which boosts convergence speed by leveraging the best individuals to guide the search.

These enhancements significantly improve the balance between exploration and exploitation, ensuring high-quality solutions and optimal global optima. As evidenced by the results in Table 4, EBMLO-DBO is a highly promising algorithm in the field of optimization, outperforming several well-established algorithms. Its superior ability to solve global optimization tasks makes it an invaluable tool for complex optimization problems.

### Convergence behavior analysis

**Fig. 7:**
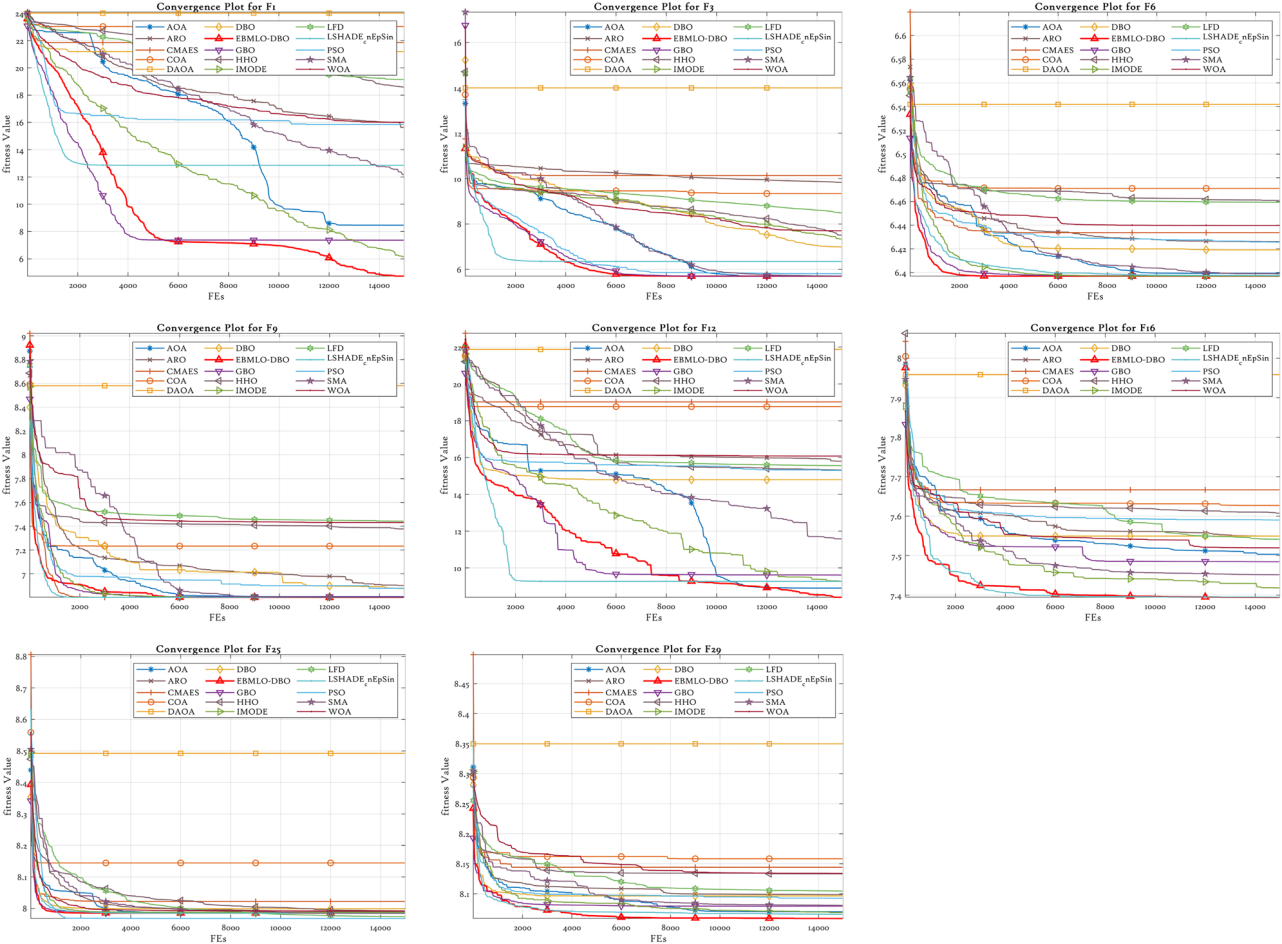
Partial convergence curves for CEC’17 with D = 10.

**Fig. 8:**
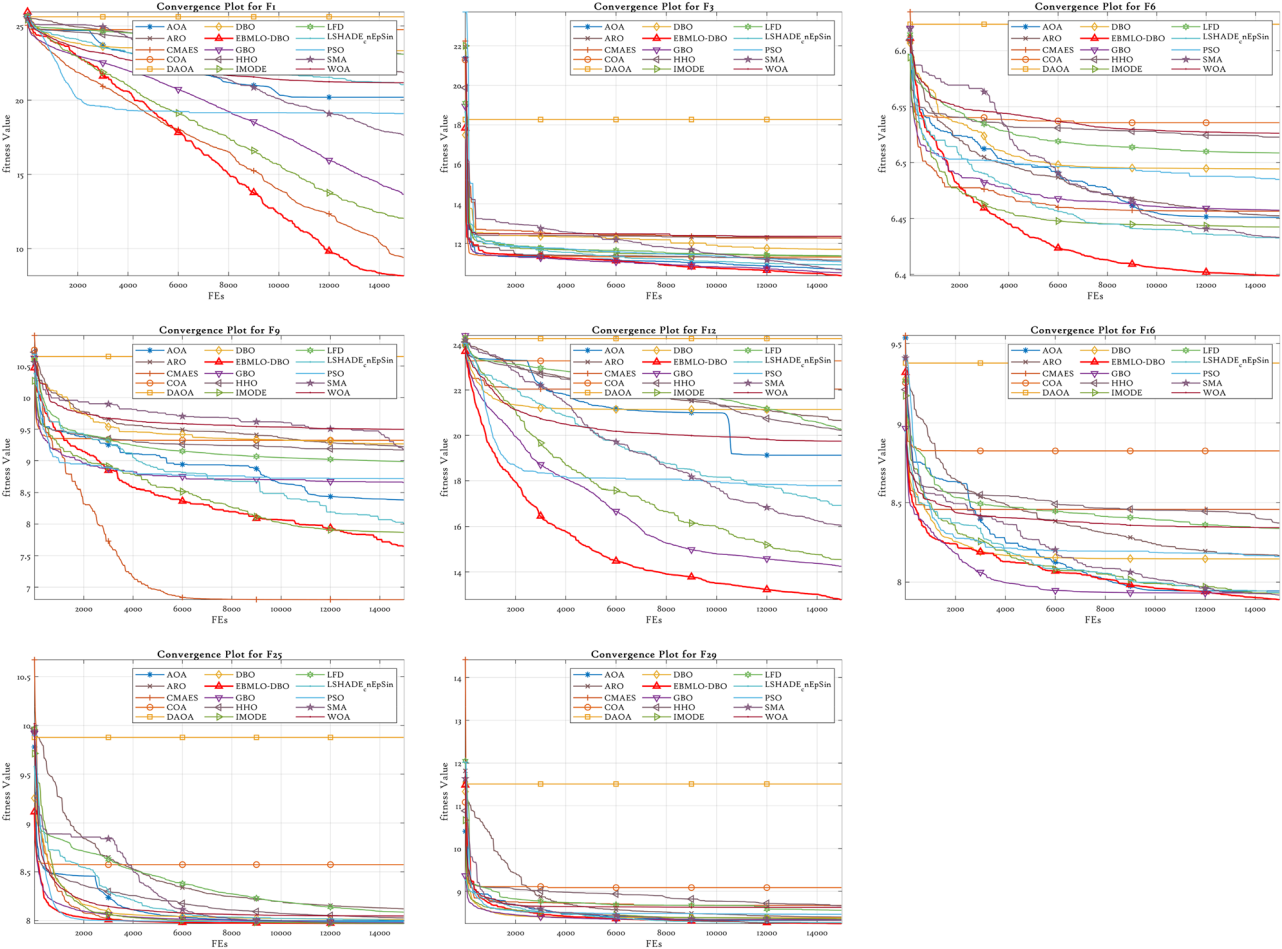
Partial convergence curves for CEC’17 with D = 30.

**Fig. 9:**
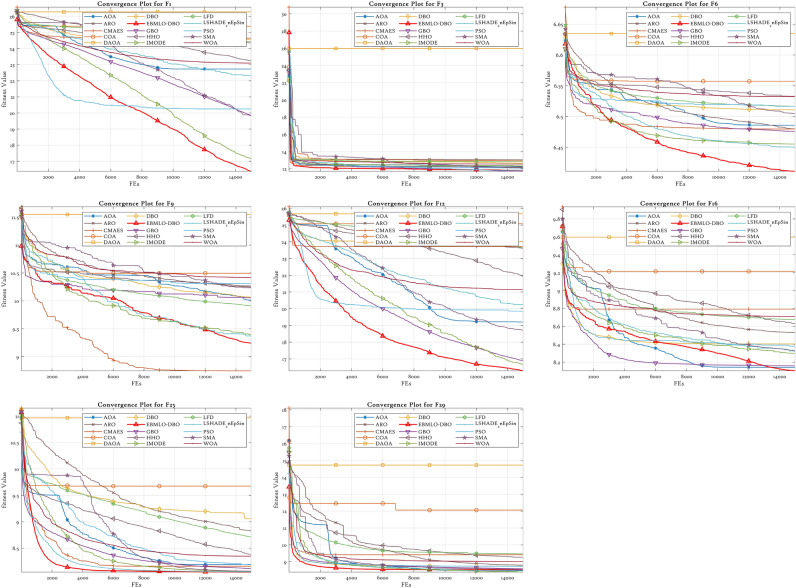
Partial convergence curves for CEC’17 with D = 50.

**Fig. 10:**
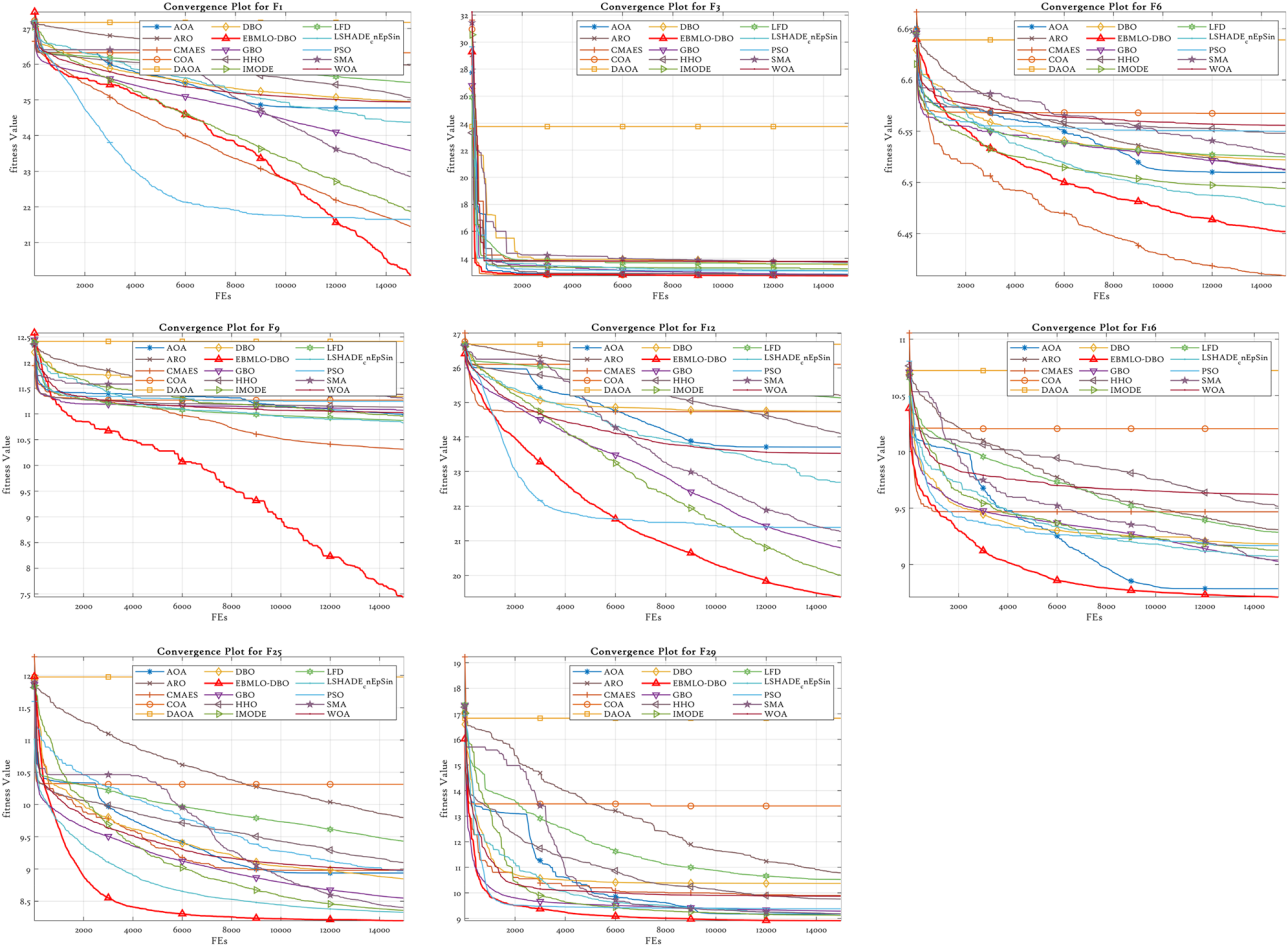
Partial convergence curves for CEC’17 with D = 100.

This section examines the convergence behavior and scalability performance of the EBMLO-DBO algorithm compared to eleven state-of-the-art optimization algorithms across varying dimensional complexities, utilizing selected benchmark functions from different categories of the CEC’17 test suite to evaluate algorithmic robustness and effectiveness as problem dimensionality increases from 10 to 100 dimensions^105^. These results are shown in Figs. 7, 8, 9, 10

The convergence behavior analysis across varying dimensional complexities from 10 to 100 dimensions reveals the robust scalability and superior performance characteristics of the EBMLO-DBO algorithm compared to eleven state-of-the-art optimization algorithms including AOA, ARO, CMAES, COA, DBO, GBO, HHO, IMODE, LFD, LSHADE_nEpSin, PSO, SMA, and WOA. The comprehensive evaluation conducted using selected benchmark functions from different categories of the CEC’17 test suite demonstrates that EBMLO-DBO consistently maintains its competitive advantage across all dimensional settings, with particularly notable performance improvements becoming more pronounced as problem dimensionality increases. In the 10-dimensional setting, EBMLO-DBO exhibits superior convergence characteristics across all function categories, achieving the lowest fitness values in unimodal functions F1 and F3, where it demonstrates rapid initial descent followed by stable convergence, while competitors like DBO, DAOA, and COA show premature stagnation or oscillatory behavior. The multimodal functions F6 and F9 reveal EBMLO-DBO’s enhanced exploration capabilities, as it successfully navigates complex landscapes with multiple local optima, consistently outperforming algorithms such as PSO, GBO, and WOA that frequently become trapped in suboptimal regions.

As dimensionality increases to 30 dimensions, the performance gap between EBMLO-DBO and competing algorithms becomes more pronounced, particularly evident in the unimodal function F1 where EBMLO-DBO achieves remarkable fitness improvements from approximately 26 to below 8, while most competitors struggle to achieve comparable convergence rates. The hybrid functions F12 and F19 in 30 dimensions showcase EBMLO-DBO’s adaptive nature, maintaining smooth convergence trajectories while algorithms like ARO, SMA, and CMAES exhibit irregular convergence patterns with frequent plateaus and limited improvement in later iterations. The composite functions F25 and F29 present the most challenging optimization scenarios in 30 dimensions, yet EBMLO-DBO demonstrates exceptional stability, achieving consistent convergence to near-optimal solutions while maintaining computational efficiency throughout the optimization process. The 50-dimensional results further emphasize EBMLO-DBO’s scalability advantages, where the algorithm continues to exhibit superior performance across all function categories, with particularly impressive results in multimodal function F9 where it achieves convergence to approximately 9.2 compared to competitor algorithms that remain above 10.5, and in hybrid function F12 where EBMLO-DBO reaches fitness values below 17 while most alternatives plateau above 20.

The 100-dimensional analysis represents the most challenging optimization environment, where the curse of dimensionality typically causes significant performance degradation in traditional optimization algorithms, yet EBMLO-DBO maintains its superior convergence characteristics with remarkable consistency. In the 100-dimensional unimodal function F1, EBMLO-DBO demonstrates exceptional scalability by achieving continuous improvement from initial values to final fitness values, while competing algorithms like DBO, DAOA, and ARO show minimal improvement or complete stagnation after early iterations. The multimodal functions F6 and F9 in 100 dimensions reveal EBMLO-DBO’s robust exploration–exploitation balance, where it consistently achieves the lowest fitness values across all evaluation points, with F9 showing particularly dramatic improvements, significantly outperforming all competitors that struggle to achieve fitness values below 10. The hybrid and composite functions F12, F19, F25, and F29 in 100 dimensions demonstrate EBMLO-DBO’s ability to handle the most complex optimization landscapes, maintaining smooth convergence trajectories and achieving superior final solutions, while most competing algorithms exhibit premature convergence, irregular oscillations, or complete stagnation. The consistent performance superiority across all dimensional settings can be attributed to the synergistic integration of the four enhancement strategies within EBMLO-DBO, where the Bernoulli map initialization ensures diverse starting points that become increasingly crucial as dimensionality increases, the ELS strategy provides intelligent guidance that scales effectively with problem complexity, the MWM maintains exploration capabilities essential for high-dimensional escape from local optima, and the LEO enhances exploitation effectiveness that becomes increasingly important for fine-tuning solutions in complex high-dimensional spaces.

### Statistical analysis of EBMLO-DBO

To establish the statistical significance of the EBMLO-DBO algorithm’s performance superiority, the Wilcoxon signed-rank test was implemented as a non-parametric statistical analysis method to determine whether the proposed EBMLO-DBO demonstrates statistically meaningful differences compared to competing optimization algorithms^106^. This statistical validation was conducted through 30 independent experimental runs for each comparative algorithm across the comprehensive benchmark function suite, ensuring robust statistical reliability and eliminating potential bias from random variations. The Wilcoxon signed-rank test was executed with a confidence level of 95% (α = 0.05) to evaluate the statistical significance of performance differences between the solution quality achieved by EBMLO-DBO and that obtained by each competitor algorithm. The comprehensive statistical analysis results are systematically organized and documented in Tables 5 and A1 for detailed examination. Following standard statistical hypothesis testing protocols, when the computed p-value falls below the threshold of 0.05, the null hypothesis of no significant difference is rejected, thereby confirming the existence of statistically significant performance distinctions between the compared algorithms. Conversely, when the p-value exceeds 0.05, the performance differences between the two algorithmic approaches are considered statistically insignificant. The results presented in Table 5 provide conclusive evidence that the EBMLO-DBO algorithm exhibits statistically significant performance improvements when compared to all benchmark algorithms including WOA, NOA, COA, HHO, PSO, DBO, GBO, AOA, SMA, ARO, and LFD, thereby establishing the statistical validity of EBMLO-DBO’s superior optimization capabilities. Furthermore, the table demonstrates the statistical differentiation between EBMLO-DBO and other advanced optimization methodologies, confirming that EBMLO-DBO maintains statistically significant advantages across most benchmark functions, thus validating the robustness and reliability of the proposed algorithmic enhancements.

**Table 5:** Statistical analysis based on Wilcoxon test between EBMLO-DBO and its rivals using CEC2022, D = 20.

F	AOA	DBO	LFD	SMA	PSO	GBO	ARO	HHO	NOA	WOA	COA
C22-G1	1.734398LEL-06	1.734398LEL-06	1.734398LEL-06	1.734398LEL-06	1.734398LEL-06	1.734398LEL-06	1.734398LEL-06	1.734398LEL-06	1.734398LEL-06	1.734398LEL-06	1.734398LEL-06
C22-G2	2.353421LEL-06	1.734398LEL-06	1.479542LEL-02	1.149922LEL-04	1.149922LEL-04	1.734398LEL-06	1.734398LEL-06	1.238080LEL-05	8.589583LEL-02	4.285686LEL-06	1.359477LEL-04
C22-G3	1.734398LEL-06	2.830789LEL-04	3.515237LEL-06	4.285686LEL-06	2.603328LEL-06	1.734398LEL-06	1.734398LEL-06	1.734398LEL-06	1.734398LEL-06	1.734398LEL-06	1.734398LEL-06
C22-G4	1.734398LEL-06	7.690859LEL-06	3.709353LEL-01	1.734398LEL-06	1.734398LEL-06	2.802144LEL-01	1.734398LEL-06	7.157034LEL-04	1.734398LEL-06	2.603328LEL-06	1.734398LEL-06
C22-G5	1.734398LEL-06	2.105260LEL-03	1.734398LEL-06	7.513662LEL-05	1.149922LEL-04	7.521331LEL-02	1.734398LEL-06	1.734398LEL-06	1.734398LEL-06	1.734398LEL-06	1.920921LEL-06
C22-G6	1.734398LEL-06	3.609433LEL-03	1.734398LEL-06	2.255124LEL-03	4.729202LEL-06	5.287248LEL-04	1.734398LEL-06	2.843424LEL-05	2.126636LEL-06	1.734398LEL-06	1.359477LEL-04
C22-G7	1.734398LEL-06	5.751653LEL-06	6.319757LEL-05	4.729202LEL-06	3.724265LEL-05	1.113801LEL-03	1.734398LEL-06	8.918727LEL-05	1.734398LEL-06	4.729202LEL-06	2.584559LEL-03
C22-G8	1.734398LEL-06	4.449337LEL-05	2.353421LEL-06	9.315659LEL-06	1.742281LEL-04	5.706437LEL-04	1.920921LEL-06	2.878599LEL-06	1.734398LEL-06	1.734398LEL-06	1.359477LEL-04
C22-G9	1.920921LEL-06	4.389618LEL-03	1.734398LEL-06	1.734398LEL-06	1.734398LEL-06	7.271050LEL-03	1.734398LEL-06	1.734398LEL-06	1.734398LEL-06	1.734398LEL-06	1.734398LEL-06
C22-G10	4.449337LEL-05	1.734398LEL-06	7.271050LEL-03	1.734398LEL-06	1.149922LEL-04	4.071512LEL-05	7.712174LEL-04	1.734398LEL-06	4.860261LEL-05	2.126636LEL-06	3.112315LEL-05
C22-G11	1.734398LEL-06	4.285686LEL-06	1.734398LEL-06	2.224827LEL-04	3.112315LEL-05	3.882182LEL-06	1.024633LEL-05	2.596713LEL-05	6.983783LEL-06	3.515237LEL-06	9.753872LEL-01
C22-G12	1.734398LEL-06	1.734398LEL-06	1.734398LEL-06	3.515237LEL-06	1.734398LEL-06	1.656553LEL-02	1.734398LEL-06	1.734398LEL-06	1.734398LEL-06	1.734398LEL-06	1.734398LEL-06

**Fig. 11:**
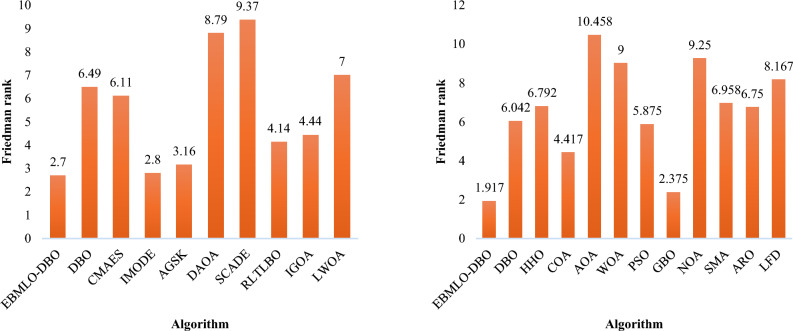
Friedman rank analysis for the EBMLO-DBO and its rivals using different test functions.

In addition, the Friedman test was used to rank each optimization algorithm, and the test findings are provided in Fig. 11. Critically looking at the data of Fig. 11 reveals that the improved dung beetle algorithm EBMLO-DBO has a lower average rating than the other algorithms. This means that the effectiveness of the augmentation can be established. In other words, the figure shows the Friedman rank test results for CEC’17-D50, compared with advanced algorithms and CEC’22-D20 compared to a set of classical and recent algorithms which provides an overall summary of the ranks for different occurrences and dimensions of the problem. It is clear that for both comparisons the EBMLO-DBO is the best by obtaining the lowest Friedman rank and is ranked the first. This indicates that EBMLO-DBO is a powerful promising algorithm that can handle different types of functions with different dimensions this is due to the proposed improvements that enhanced the solution quality, accuracy, and convergence speed of DBO toward the best global solutions.

## PV parameter estimation using EBMLO-DBO

The process of determining parameters for PV models can be modeled as an optimization problem. As previously mentioned, minimizing the disparity between simulated data and experimental data is the main objective of this optimization problem. Hence, the objective function in this case can be formulated as computing the disparity between the manufacture measured current and the estimated current. Subsequently, optimization approaches are employed to investigate and ascertain the parameter values minimizing this disparity. The absolute difference between the expected and observed current is defined as the individual absolute current error (AEC) which can be formulated in Eq. (37) and (38) for both the single diode and double diode, respectively:

37 $$AEC={I}_{PC}-{I}_{rsD}\left({e}^{\frac{Q\left({V}_{l}+{I}_{\text{out }}{R}_{se}\right)}{(NKTT)}}-1\right)-\frac{\left({V}_{i}+{I}_{\text{out }}{R}_{se}\right)}{\left({R}_{SH}\right)}-{I}_{\text{out}}$$

38 $$\begin{array}{l}AEC={I}_{PC}-{I}_{rsD1}\left({e}^{\frac{Q\left({V}_{l}+{I}_{\text{out }}{R}_{se}\right)}{(N1KT)}}-1\right)-{I}_{rsD2}\left({e}^{\frac{Q\left({V}_{V}+{I}_{\text{out }}{R}_{se}\right)}{(N2KT)}}-1\right)\\ -\frac{\left({V}_{l}+{I}_{\text{out }}{R}_{se}\right)}{\left({R}_{sH}\right)}-{I}_{\text{out}}\end{array}$$

Moreover, the root mean square error (RMSE) is computed by squaring the average of the observed and predicted current values, and then taking the square root of this value. This term represents the cumulative discrepancy between the two sets of data:

39 $$RME(x)=\sqrt{\frac{1}{N}{\sum }_{1}^{N} AE{C}^{2}}$$

where $N$ denotes the entire quantity of data points that have been measured. The vector $x$ comprises the parameter values ascertained by the optimization approach. The variables included in the single-diode model, denoted by $x$, are ${I}_{rsD}$, ${I}_{PC}$, $N$, ${R}_{se}$ and ${R}_{SH}$. On the other hand, the double-diode model contains the variables ${I}_{PC}$, ${I}_{rsD1}$, ${I}_{rsD2}$, ${R}_{SH}$, ${R}_{se}$, ${N}_{1}$, and ${N}_{2}$.

### Experimental settings and comparative algorithms

An ideal scenario to exhibit the effectiveness of the proposed method is to illustrate its ability to precisely represent the properties of PV module models, including the double-diode, PV module, single-diode models. The data from^107^ were selected as benchmarks because to their widespread usage in evaluating various methods for predicting parameters of photovoltaic models. The investigations involved the serial connection of 36 polycrystalline PV cells with a commercial silicon PV cell from RTC France, which had a diameter of 57 mm. The experiments were carried out at an irradiation level of 1000 W/m^2^ and a 33 °C temperature. Furthermore, a monocrystalline STM6-40/36 module underwent testing under specific conditions, including an irradiation level of 1000 W/m^2^ and a temperature of 45 °C. Previous research has utilized parameter ranges that ensure a reliable search space for all problems. The parameter ranges for PV cells and modules are illustrated in Table 6.

**Table 6:** Parameter ranges for the three different PV models.

Parameters	Single/Double diode Upper	Single/Double diode Lower	PV module Upper	PV module Lower
${I}_{pc}$,(Ampere)	1	0	2	0
${I}_{rsD}$, ${I}_{rsD1}$, ${I}_{rsD2}$,( mu Ampere)	1	0	50	0
${R}_{se}$,(Ohm)	0.5	0	2	0
${R}_{sH}$,(Ohm)	100	0	2000	0
$N, N1, N2$	2	1	50	1

A wide range of approaches has been engaged for estimating the parameters of the PV models, including but not limited to the analytical heuristic and deterministic approaches^108^. Analytical procedures are widely adopted due to their practicality and ease of use^109^. In addition, the results cannot always be reliable or precise enough since the basic assumptions used may be too general^110^. In deterministic approaches, the estimation is bonded by the assumptions made within the model and may be caught up in local maxima. To be certain, the convexity and differentiability of the model can guide the efficiency of the models especially for multi-modal nonlinear PV models. On the contrary, the heuristic approaches often outperform the analytical and deterministic methods in terms of performance. These are flexible methods that do not impose constraints on the problem at hand and are therefore suitable for a wide range of practical problems. Heuristics approaches have indeed appeared as promising alternatives when it comes to the estimation of parameters in PV models.

The proposed EBMLO-DBO is tested using the recently developed classical and advanced algorithms to overcome the complexity of parameters extraction for different solar systems. The approaches encompassed are RTLBO^111^, SSA^112^, IJAYA^113^, BES^114^, CGO-LS^115^, MLBSA ^116^, NOA^64^, GBO^83^, and DBO. Since swarm intelligence algorithms are non-deterministic, all runs are made with the same setting. In fair comparisons, the maximum iteration number of each approach is set to 500 for all experiments. For each problem, the process repeats 30 independent times in order to ensure statistically valid comparisons. The accuracy and robustness of the solution obtained by each scheme are measured with the statistical measure. After that, a deeper comparison is drawn based on the best RMSE values obtained using 30 runs in each method. The smallest magnitude of the RMSE values is highlighted in bold.

### Results analysis

In this section, the proposed EBMLO-DBO is compared with its competitors for all three PV models: double-diode model, single-diode model, and PV module model. I-V and P–V curves are shown for this experiment. In addition, the estimated parameters and the statistical metrics for each model are presented in a comprehensive manner.

**Fig. 12:**
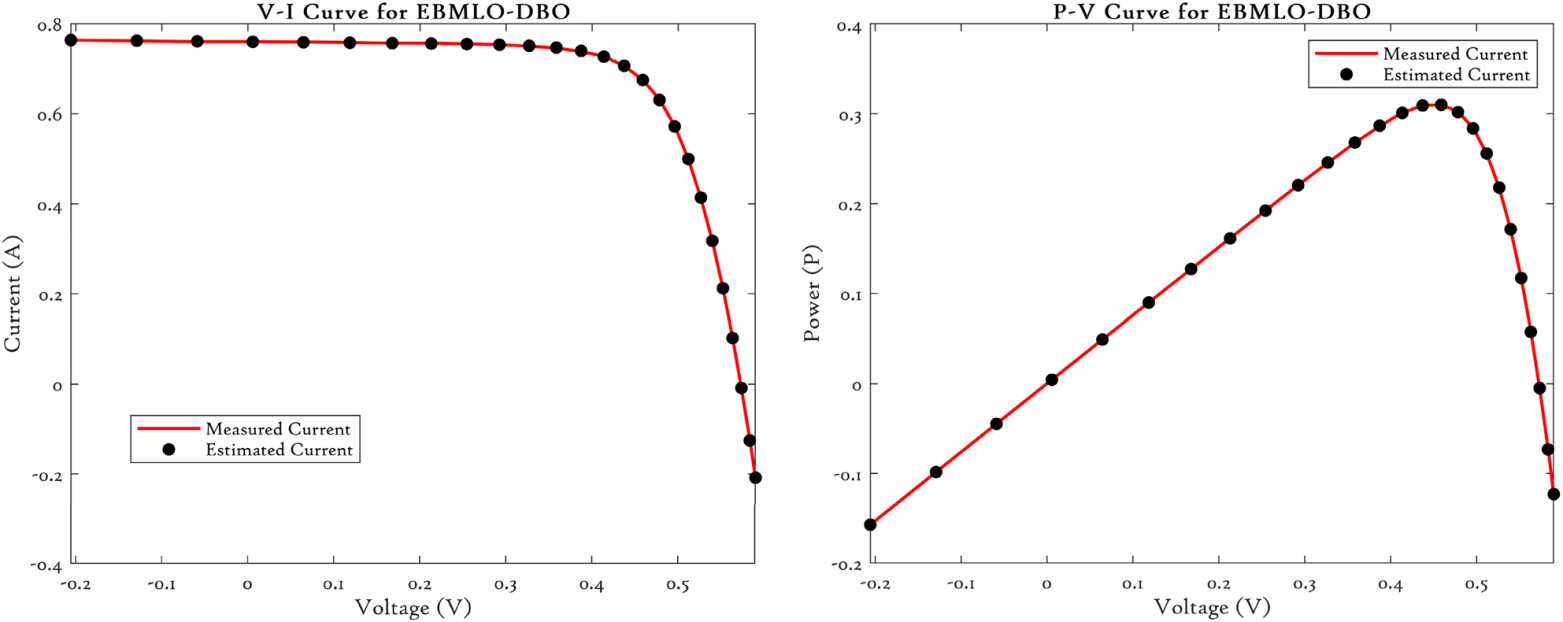
The V–I and P–V curves for single diode model based on EBMLO-DBO.

**Fig. 13:**
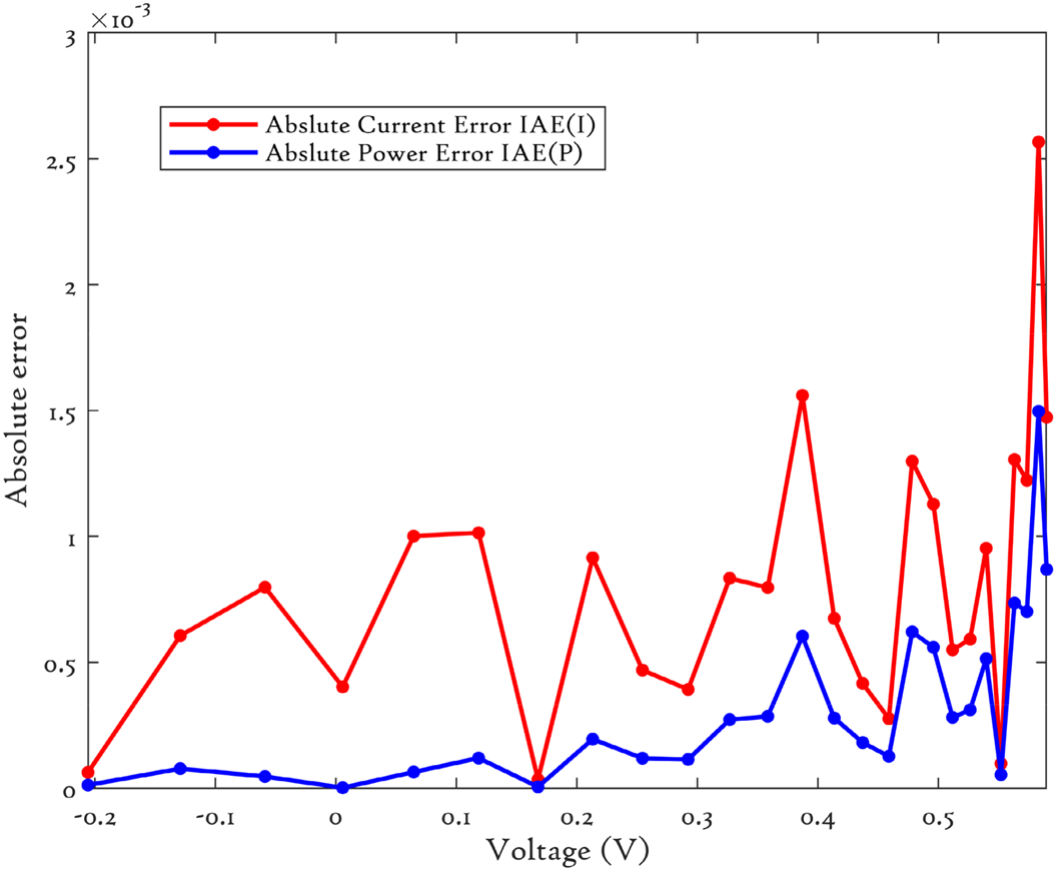
The IAE comparisons on single diode model using EBMLO-DBO.

*Regarding to the single diode model:* The EBMLO-DBO algorithm accurately predicted the current–voltage (I-V) and power-voltage (P–V) curves for the Single Diode Model (SDM), as shown in Fig. 12. The results obtained via EBMLO-DBO exhibit a high degree of correlation with the experimental data across the whole range of voltages. Figure 13 illustrates the differences, in absolute error, between the values obtained from experiments and those obtained from simulations. Table 7 displays the recorded current measurements acquired during a simulated test session. The computed values for the Individual Absolute Error (IAE) are included in the analysis. These values are determined using the following Equation:

**Table 7:** The absolute error based on EBMLO-DBO for single diode model.

Component	Measured Voltage	Measured Current	Estimated Current ${I}_{est}$	$IAE\left({I}_{est}\right)$	Estimated Power $({P}_{est})$	$IAE({P}_{est})$
1	-0.2057	0.764	0.7640633	6.33E-05	-0.157167821	1.3E-05
2	-0.1291	0.762	0.7626063	0.0006063	-0.098452473	7.83E-05
3	-0.0588	0.7605	0.7612983	0.0007983	-0.04476434	4.69E-05
4	0.0057	0.7605	0.7600973	0.0004027	0.004332555	2.3E-06
5	0.0646	0.76	0.7589983	0.0010017	0.04903129	6.47E-05
6	0.1185	0.759	0.7579853	0.0010147	0.089821258	0.00012
7	0.1678	0.757	0.7570353	3.53E-05	0.127030523	5.92E-06
8	0.2132	0.757	0.7560843	0.0009157	0.161197173	0.000195
9	0.2545	0.7555	0.7550303	0.0004697	0.192155211	0.00012
10	0.2924	0.754	0.7536073	0.0003927	0.220354775	0.000115
11	0.3269	0.7505	0.7513343	0.0008343	0.245611183	0.000273
12	0.3585	0.7465	0.7472973	0.0007973	0.267906082	0.000286
13	0.3873	0.7385	0.7400603	0.0015603	0.286625354	0.000604
14	0.4137	0.728	0.7273253	0.0006747	0.300894477	0.000279
15	0.4373	0.7065	0.7069163	0.0004163	0.309134498	0.000182
16	0.459	0.6755	0.6752233	0.0002767	0.309927495	0.000127
17	0.4784	0.632	0.6307013	0.0012987	0.301727502	0.000621
18	0.496	0.573	0.5718713	0.0011287	0.283648165	0.00056
19	0.5119	0.499	0.4995503	0.0005503	0.255719799	0.000282
20	0.5265	0.413	0.4135923	0.0005923	0.217756346	0.000312
21	0.5398	0.3165	0.3174533	0.0009533	0.171361291	0.000515
22	0.5521	0.212	0.2120983	9.83E-05	0.117099471	5.43E-05
23	0.5633	0.1035	0.1021943	0.0013057	0.057566049	0.000736
24	0.5736	-0.01	-0.0087767	0.0012233	-0.005034315	0.000702
25	0.5833	-0.123	-0.1255667	0.0025667	-0.073243056	0.001497
26	0.59	-0.21	-0.2085267	0.0014733	-0.123030753	0.000869

40 $$IAE=\left|{I}_{\text{measure }}-{I}_{\text{simulate}}\right|$$

The results indicate that the IAE values are below 0.0025667. This demonstrates that the single-diode model, when simulated using the suggested algorithm, closely corresponds to the real features of solar cells. In addition, the EBMLO-DBO algorithm has superior performance compared to other approaches, including RTLBO, SSA, IJAYA, BES, CGO-LS, MLBSA, GBO, and DBO. This is clearly seen in Table 8, which provides detailed information on the five parameter values and the corresponding RMSE. In addition, Table 9 clearly shows that both the proposed EBMLO-DBO and algorithm RTLBO consistently produced the lowest RMSE value of 9.8602E-4 in both circumstances. These findings indicate that the suggested technique is very efficient in determining the parameters of the SDM.

**Fig. 14:**
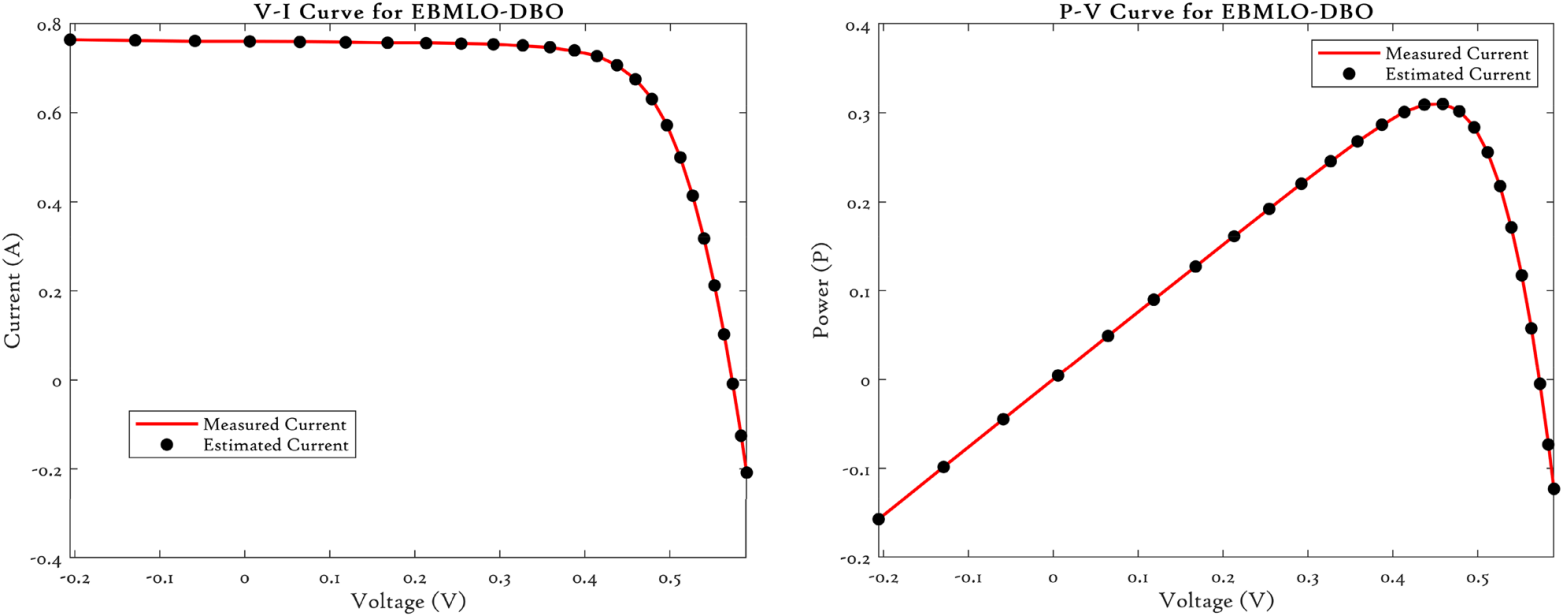
The V–I and P–V curves for double diode model based on EBMLO-DBO.

**Fig. 15:**
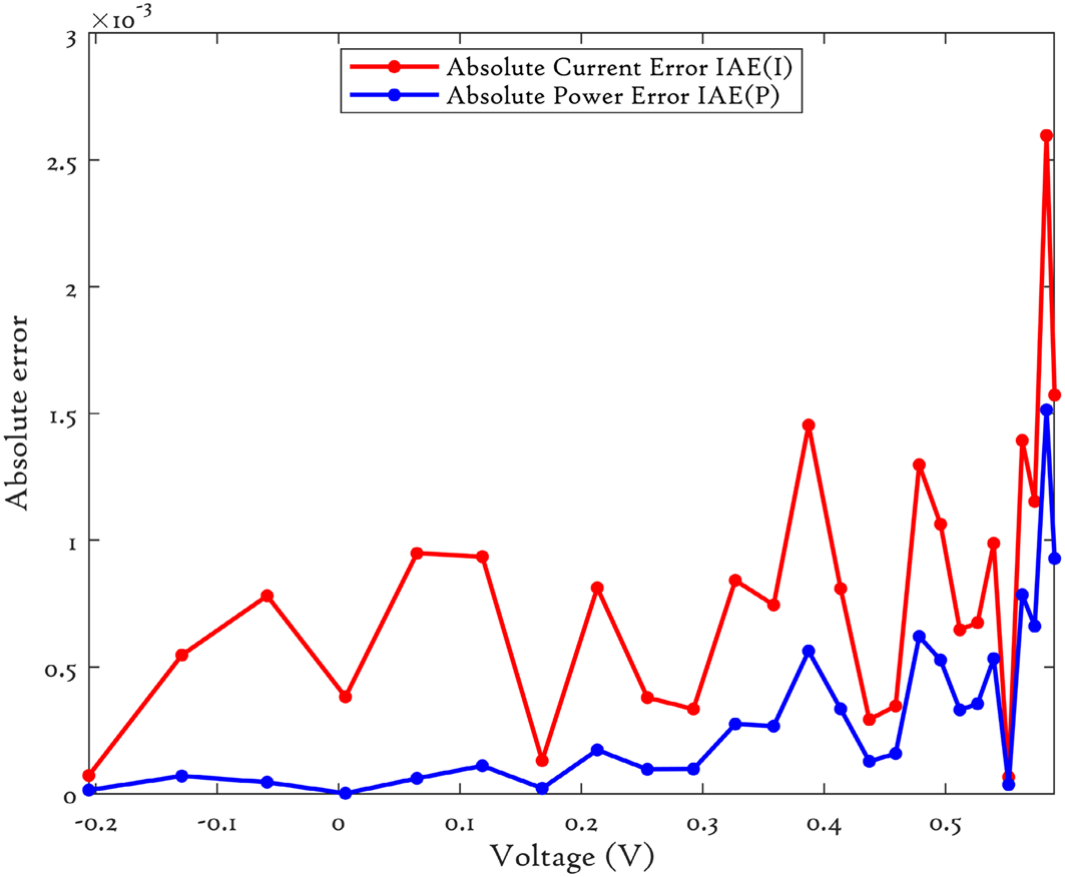
The IAE comparisons on double diode model using EBMLO-DBO.

**Fig. 16:**
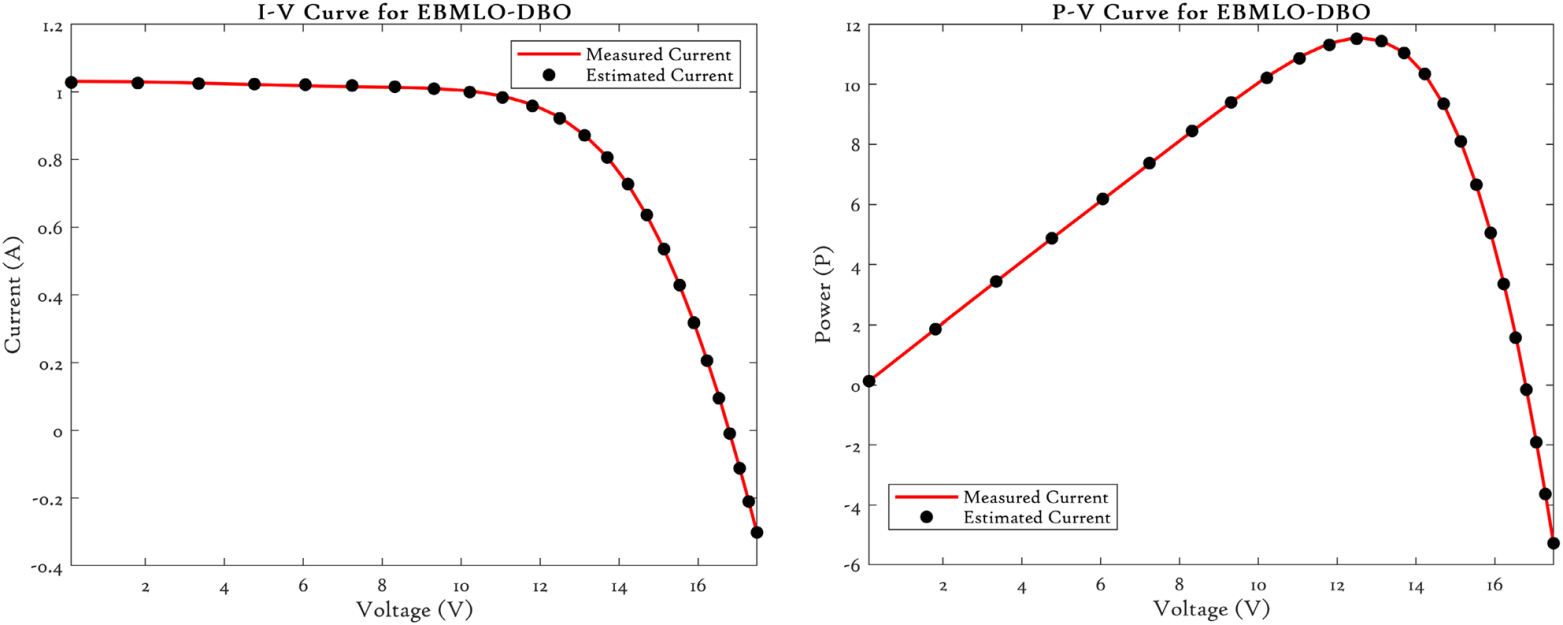
The V–I and P–V curves for double diode model based on EBMLO-DBO.

**Fig. 17:**
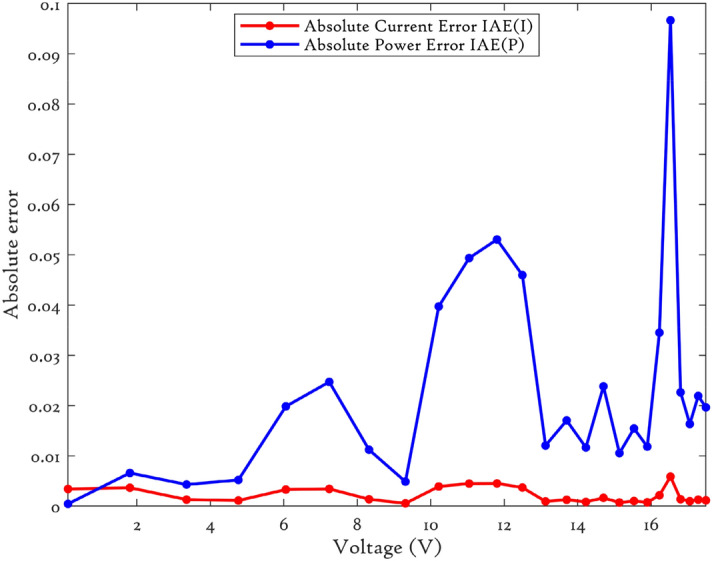
The IAE comparisons on double diode model using EBMLO-DBO.

- *Regarding the result analysis of double diode model:* Fig. 14 presents a juxtaposition of the estimated characteristics generated by EBMLO-DBO and the factual measured data. In addition, Fig. 15 visually illustrates the IAE for the double-diode module. The simulations demonstrate a close resemblance between the simulated I-V and P–V characteristic curves and the experimental data. Table 9 presents the power, current and IAE parameters of the simulation. At every voltage measurement point, the IAE is lower than 0.002597, and the. This indicates that the double-diode module designed by EBMLO-DBO effectively represents the key properties of solar cells. Furthermore, Table 10 displays a juxtaposition of the outcomes achieved by EBMLO-DBO in relation to those of comparative algorithms. The table provided contains a comparison of the RMSE as well as the values of the seven retrieved parameters. Out of the seven ways that were assessed, the proposed EBMLO-DBO algorithm obtains the lowest RMSE value.
- *Regarding the result analysis of PV module mode:* Fig. 16 presents the I-V and P–V curves for the Photowatt-PWP 201 module model, demonstrating a strong correlation between the measured and simulated data, indicating a high level of consistency. Figure 17 displays the measured and simulated currents for the IAE of the PV module model. In addition, Table 11 provides comprehensive information on the power and current values obtained from this experiment, as well as the accompanying IAE. The IAE value is explicitly stated to be lower than 0.00585036. These results indicate that the PV module model parameters advised by EBMLO-DBO are dependable. Estimating all five parameters that characterize the PV module model is essential. Table 12 presents the extracted values for these five parameters, along with the optimal RMSE value achieved by seven algorithms across 30 tests. A summary of this data is provided in Table 13, showing that EBMLO-DBO achieves the highest performance in terms of RMSE. Therefore, it can be concluded that the proposed EBMLO-DBO method excels in parameter estimation for the PV module model, outperforming other approaches.

**Table 8:** Comparative analysis between EBMLO-DBO and other algorithms for single diode model.

	${I}_{ph}$	${I}_{sd}$	${R}_{s}$	${R}_{sh}$	$N$	$RMSE$
EBMLO-DBO	0.76002740400	0.00000031884	0.03589892940	53.56881870000	1.47376373000	**0.0009686818779**
DBO	0.76008259900	0.00000029366	0.03622674520	51.37125310000	1.46563466000	0.0009808452770
RTLBO	0.76002924800	0.00000031845	0.03590367100	53.52402020000	1.47364150000	0.0009686848538
SSA	0.75990837800	0.00000032539	0.03575123680	53.55578580000	1.47581292000	0.0009790186799
IJAYA	0.76002685000	0.00000031925	0.03589376920	53.59689310000	1.47388998000	0.0009686848927
BES	0.75961275200	0.00000054042	0.03433995880	95.85532600000	1.52802054000	0.0009686914521
CGO-LS	0.75996312800	0.00000034432	0.03559072430	55.87040790000	1.48144794000	0.0009794534554
NOA	0.76002052300	0.00000032011	0.03588439280	53.74259370000	1.47415668000	0.0009687165785
GBO	0.76002833900	0.00000031807	0.03590897100	53.51105620000	1.47352167000	0.0009686928320

**Table 9:** The absolute error based on EBMLO-DBO for double diode model.

	Measured Voltage	Measure Current	Estimated current $({I}_{est})$	$IAE\left({I}_{est}\right)$	Estimated Power $({P}_{est})$	$IAE({P}_{est})$
1	-0.2057	0.764	0.763927	7.27E-05	-0.15714	1.49544E-05
2	-0.1291	0.762	0.762547	0.000547	-0.09844	7.06564E-05
3	-0.0588	0.7605	0.761281	0.000781	-0.04476	4.59404E-05
4	0.0057	0.7605	0.760117	0.000383	0.004333	2.18139E-06
5	0.0646	0.76	0.759051	0.000949	0.049035	6.1286E-05
6	0.1185	0.759	0.758064	0.000936	0.089831	0.00011088
7	0.1678	0.757	0.757131	0.000131	0.127047	2.20321E-05
8	0.2132	0.757	0.756187	0.000813	0.161219	0.000173268
9	0.2545	0.7555	0.75512	0.00038	0.192178	9.66337E-05
10	0.2924	0.754	0.753665	0.000335	0.220372	9.78663E-05
11	0.3269	0.7505	0.751342	0.000842	0.245614	0.000275348
12	0.3585	0.7465	0.747245	0.000745	0.267887	0.00026719
13	0.3873	0.7385	0.739954	0.001454	0.286584	0.00056325
14	0.4137	0.728	0.72719	0.00081	0.300839	0.000334973
15	0.4373	0.7065	0.706793	0.000293	0.309081	0.00012826
16	0.459	0.6755	0.675153	0.000347	0.309895	0.000159135
17	0.4784	0.632	0.630702	0.001298	0.301728	0.00062082
18	0.496	0.573	0.571936	0.001064	0.28368	0.000527595
19	0.5119	0.499	0.499647	0.000647	0.255769	0.000331353
20	0.5265	0.413	0.413675	0.000675	0.2178	0.000355545
21	0.5398	0.3165	0.317488	0.000988	0.17138	0.000533484
22	0.5521	0.212	0.212066	6.63E-05	0.117082	3.66042E-05
23	0.5633	0.1035	0.102106	0.001394	0.057516	0.000785071
24	0.5736	-0.01	-0.00885	0.001153	-0.00507	0.000661533
25	0.5833	-0.123	-0.1256	0.002597	-0.07326	0.001514655
26	0.59	-0.21	-0.20843	0.001573	-0.12297	0.000928247

**Table 10:** Comparative analysis between EBMLO-DBO and other algorithms for double diode model.

	${I}_{ph}$	${I}_{sd1}$	${I}_{sd2}$	${R}_{s}$	${R}_{sh}$	${N}_{1}$	${N}_{2}$	$RMSE$
EBMLO-DBO	0.760593391	0.000000725	0.035753187	55.468570800	1.912381590	0.000000223	1.439677542	**0.000981307**
DBO	0.760631048	0.000000170	0.036036241	54.968555900	1.349438550	0.000000967	1.988116632	0.000984829
RTLBO	0.760564308	0.000000270	0.035568294	55.098982700	1.682355260	0.000000225	1.443516512	0.000984083
SSA	0.760599729	0.000000145	0.035605818	53.651758200	1.620933310	0.000000239	1.448515232	0.000984913
IJAYA	0.760592725	0.000000265	0.035514105	54.241884800	1.383355410	0.000000260	1.988525692	0.000982878
BES	0.762749970	0.000000159	0.034606503	47.780488600	1.361982910	0.000000418	1.667467632	0.001641912
CGO-LS	0.760581653	0.000000362	0.034690241	58.047895300	1.411102630	-0.000000003	1.777386502	0.001039050
NOA	0.760582585	0.000000302	0.035385478	53.804415000	1.393756960	-0.000000003	1.850726562	0.000984854
GBO	0.760589975	0.000000301	0.035387577	53.731147200	1.393689780	-0.000000003	1.897266112	0.000984848

**Table 11:** The absolute error based on EBMLO-DBO for double diode model.

	Measured Voltage	Measured Current	Estimated Current ${I}_{est}$	$IAE\left({I}_{est}\right)$	Estimated Power $({P}_{est})$	$IAE({P}_{est})$
1	0.1248	1.0315	1.02810164	0.00339836	0.12830708	0.00042412
2	1.8093	1.03	1.02636364	0.00363636	1.85699973	0.00657927
3	3.3511	1.026	1.02472464	0.00127536	3.43395474	0.00427386
4	4.7622	1.022	1.02308964	0.00108964	4.87215748	0.00518908
5	6.0538	1.018	1.02127464	0.00327464	6.18259242	0.01982402
6	7.2364	1.0155	1.01891364	0.00341364	7.37326666	0.02470246
7	8.3189	1.014	1.01534564	0.00134564	8.44655884	0.01119424
8	9.3097	1.01	1.00947864	0.00052136	9.39794329	0.00485371
9	10.2163	1.0035	0.99961164	0.00388836	10.21233240	0.03972465
10	11.0449	0.988	0.98353064	0.00446936	10.86299757	0.04936363
11	11.8018	0.963	0.95850464	0.00449536	11.31208006	0.05305334
12	12.4929	0.9255	0.92182164	0.00367836	11.51622557	0.04595338
13	13.1231	0.8725	0.87158264	0.00091736	11.43786614	0.01203861
14	13.6983	0.8075	0.80625664	0.00124336	11.04434533	0.01703192
15	14.2221	0.7265	0.72731864	0.00081864	10.34399843	0.01164278
16	14.6995	0.6345	0.63612064	0.00162064	9.35065535	0.02382260
17	15.1346	0.5345	0.53519564	0.00069564	8.09997193	0.01052823
18	15.5311	0.4275	0.42849364	0.00099364	6.65497757	0.01543232
19	15.8929	0.3185	0.31775664	0.00074336	5.05007450	0.01181415
20	16.2229	0.2085	0.20637264	0.00212736	3.34796270	0.03451195
21	16.5241	0.101	0.09514964	0.00585036	1.57226217	0.09667193
22	16.7987	-0.008	-0.00934736	0.00134736	-0.15702350	0.02263390
23	17.0499	-0.111	-0.11195736	0.00095736	-1.90886179	0.01632289
24	17.2793	-0.209	-0.21026736	0.00126736	-3.63327279	0.02189909
25	17.4885	-0.303	-0.30187736	0.00112264	-5.27938221	0.01963329

**Table 12:** Comparative analysis between EBMLO-DBO and other algorithms for double diode model.

Algorithm	${I}_{ph}$	${I}_{sd}$	${R}_{s}$	${R}_{sh}$	$N$	$RMSE$
EBMLO-DBO	1.03040183	0.00000337	1.20025970	970.97129700	47.54111765	**0.00232066**
DBO	1.43548115	0.00000000	0.00000000	3.61287110	1.00000000	0.27414637
RTLBO	1.03068523	0.00000320	1.20547592	919.39200200	47.34726875	0.00232474
SSA	1.03030349	0.00000339	1.20011532	989.01516900	47.56703075	0.00232124
IJAYA	1.03040509	0.00000337	1.20026942	970.72469800	47.54095495	0.00232066
BES	1.02589257	0.00000464	1.24631121	770.07791800	48.83748745	0.01485800
CGO-LS	1.05162100	0.00000005	1.53532341	194.62042200	35.36557005	0.01102384
NOA	1.03021227	0.00000344	1.19860067	1002.25564900	47.61448905	0.00232145
GBO	1.03054767	0.00000331	1.20188363	949.55632300	47.47988505	0.00232113

**Table 13:** Statistical analysis for EBMLO-DBO and its competitors using different PV models.

Single diode Model								
	EBMLO-DBO	DBO	RTLBO	SSA	IJAYA	BES	CGO-LS	GBO
Min	**0.000969**	0.000981	0.000969	0.000979	0.000969	0.000969	0.000979	0.000969
Max	**0.000969**	0.038137	0.001016	0.001465	0.00099	0.002912	0.002153	0.002998
AVG	**9.86E-04**	2.34E-02	9.95E-04	1.14E-03	9.89E-04	2.47E-03	1.49E-03	1.12E-03
STD	**2.26E-20**	5.51E-02	1.38E-05	1.40E-04	4.73E-06	5.24E-04	3.42E-04	0.000435
Wilcoxon test significance		+	+	+	+	+	+	+
Friedman rank	**1.45**	8.09	2.48	6.21	3.24	8.52	5.23	4.25
Double diode Model								
	**EBMLO-DBO**	**DBO**	**RTLBO**	**SSA**	**IJAYA**	**BES**	**CGO-LS**	**GBO**
Min	**0.000981**	0.000985	0.000984	0.000985	0.000983	0.001642	0.001039	0.000985
Max	**9.98E-04**	3.82E-02	1.37E-03	1.49E-03	1.13E-03	4.41E-03	3.34E-02	2.08E-03
AVG	**9.88E-04**	1.25E-02	1.18E-03	1.22E-03	1.12E-03	3.32E-03	2.42E-03	1.14E-03
STD	**3.18E-06**	1.74E-02	8.12E-05	1.64E-04	5.17E-05	8.24E-04	6.17E-03	0.000259
Wilcoxon test significance		+	+	+	+	+	+	+
Friedman rank	**1.42**	6.24	2.44	6.31	3.21	8.14	5.84	4.1
PV Module Model								
	**EBMLO-DBO**	**DBO**	**RTLBO**	**SSA**	**IJAYA**	**BES**	**CGO-LS**	**GBO**
Min	**0.002321**	0.274146	0.002325	0.002321	0.002321	0.014858	0.011024	0.002321
Max	**0.002492**	0.783808	0.003074	0.002558	0.274146	0.311099	0.274146	0.441954
AVG	**2.55E-03**	3.21E-01	2.48E-03	2.51E-03	2.08E-02	1.21E-01	2.34E-01	2.92E-01
STD	**3.19E-05**	8.87E-02	1.45E-04	6.60E-05	6.91E-02	8.99E-02	9.56E-02	9.10E-02
Wilcoxon test significance		+	+	=	+	+	+	+
Friedman rank	**1.74**	8.06	3.78	2.44	3.33	7	5	6.32

The evaluation of the EBMLO-DBO algorithm’s performance employs non-parametric statistical tests and multiple metrics. Accuracy is determined by analyzing the mean and best values, while a lower worst value shows the algorithm’s capability to prevent premature convergence. Consistency is indicated by the standard deviation. The Wilcoxon signed-rank test is used to compare the proposed algorithm with baseline methods, and the Friedman test ranks the algorithms based on overall performance. A lower Friedman test rank value indicates superior performance.

In the Wilcoxon signed-rank test, different symbols represent levels of significance: a $+$ symbol means the algorithm performs worse than the proposed one at $p < 0.05$; a $-$ symbol indicates significantly better performance at $p < 0.05$; and a $=$ symbol denotes similar performance to the proposed algorithm. The results are detailed in Table 13, with the Friedman test rank values listed in the final row. Table 13 shows the statistical results for various algorithms after 30 independent trials, each consisting of 500 iterations. The analysis includes worst, best, standard deviation, average, and rank values.

Based on the data in Table 13, the Wilcoxon signed-rank test results, at a significance level of 0.05, indicate that EBMLO-DBO outperforms the other algorithms. This is shown by all $+$ signs for double and single diode models, and all $+$ signs except for EBMLO-DBO in the PV module scenario, where $=$ indicates comparable performance. Furthermore, the Friedman test supports the dominance of EBMLO-DBO, ranking it as the best algorithm for average performance of double, single, and PV modules, respectively, followed by the RTLBO and other algorithms.

Finally, the obtained results for this work ascertain that the EBMLO-DBO approach is very effective at finding the optimal photovoltaic model parameters. It presents the minimum RMSE values and outperforms complex algorithms such as RTLBO, IJaya, and DBO in terms of accuracy and speed of results. With respect to $\alpha = 0.05$, the performed Wilcoxon signed-rank test and the Friedman test proved that EBMLO-DBO enjoys a significant statistical advantage over other compared algorithms. The integration of the strategies of BM, LEO, MWM, and ELS enhances the ability of DBO to tackle complex optimization problems with several modes and local valleys. It makes this optimization algorithm versatile for a wide range of applications. This is achieved by avoiding local optima.

## Discussion

This section provides a comprehensive analysis of the experimental results obtained from EBMLO-DBO and critically evaluates its performance against existing optimization methods. The proposed EBMLO-DBO demonstrates several significant advantages over conventional optimization algorithms, particularly in terms of convergence performance and solution quality. The algorithm achieved superior Friedman ranks of 1.83 and 2.7 on CEC’22 and CEC’17 benchmark suites respectively, indicating exceptional optimization capability across diverse problem landscapes. The Bernoulli map-based initialization strategy effectively addresses premature convergence issues commonly encountered in traditional MAs by providing more diverse initial solutions, while the integration of MWM and LEO strategy enables effective escape from local optima, as evidenced by superior performance on multimodal functions.

When compared to classical algorithms such as PSO, GBO, HHO, DBO, and WOA, EBMLO-DBO shows remarkable improvements in both solution quality and consistency. Against advanced algorithms including CMAES, IMODE, LSHADE-cnEpSin and AGSK, EBMLO-DBO achieved the lowest average fitness values in 18 out of 29 CEC2017 functions, demonstrating its competitiveness with state-of-the-art methods. In photovoltaic parameter estimation applications, EBMLO-DBO consistently achieved the lowest RMSE values across all three models: single diode (9.8602E-4), double diode (9.81307E-4), and PV module (2.32066E-3), outperforming specialized algorithms like RTLBO and SSA. The ELS strategy dynamically adjusts selection probability between elite random leadership and best leadership, maintaining optimal balance throughout the optimization process and contributing significantly to the algorithm’s superior performance.

Despite these advantages, EBMLO-DBO exhibits certain limitations that warrant consideration. The integration of four enhancement strategies increases computational overhead compared to the original DBO, resulting in higher execution times. The algorithm introduces additional parameters including scaling factors, mutation probabilities, and elite group size, which require careful tuning for optimal performance across different problem domains. Memory requirements are increased due to the ELS strategy’s need to maintain separate elite and non-elite populations, which may impact scalability for large-scale problems. While effective on tested dimensions up to $D=50$, the algorithm’s performance on extremely high-dimensional problems requires further investigation. Each enhancement strategy contributes uniquely to overall performance: Bernoulli Map initialization provides an improvement in population diversity, ELS strategy effectively guides convergence but requires appropriate elite group size selection, MWM prevents premature convergence in most tested functions, and LEO significantly improves local optima escape with an increased computational overhead.

## Conclusion and future scope

This study proposed an enhanced version of the DBO algorithm, named Elite Bernoulli-based EBMLO-DBO algorithm. The main contributions of this work include the design of four strategic mechanisms—Bernoulli map-based initialization for improved population diversity, MWM for enhanced search precision, an ELS strategy to accelerate convergence, and a LEO strategy to escape local stagnation. These innovations collectively address the core weaknesses of the original DBO, particularly its slow convergence and vulnerability to premature convergence in complex landscapes.

EBMLO-DBO was rigorously validated across 41 benchmark functions from the CEC’22 and CEC’17 test suites, where it demonstrated superior performance in terms of convergence speed, solution accuracy, and robustness. The algorithm achieved Friedman rankings of 1.83 and 2.7 on the CEC’22 and CEC’17 benchmarks, respectively, outperforming many recent and classical algorithms, including PSO, HHO, COA, CMAES, and IMODE. It achieved the first rank in 50% of the CEC’22 functions and produced the best fitness values in 18 out of 29 functions on CEC’17. The statistical significance of these results was confirmed by Wilcoxon signed-rank tests, yielding p-values below 0.05 across all comparisons. In real-world scenarios, EBMLO-DBO achieved unprecedented accuracy in photovoltaic parameter estimation, producing RMSE values of 9.8602 × 10⁻^4^, 9.81307 × 10⁻^4^, and 2.32066 × 10⁻^3^ for the single-diode, double-diode, and PV module models, respectively.

Although EBMLO-DBO has proven to be highly effective, certain limitations remain. The current implementation depends on fixed hyperparameters, which may affect performance across different problem domains. The inclusion of additional operators slightly increases computational cost, and its behavior in extremely high-dimensional and dynamic environments requires further investigation.

Future research directions are categorized into immediate, medium-term, and long-term priorities to expand the capabilities and applicability of EBMLO-DBO. Immediate efforts will focus on improving scalability for problems exceeding 1000 dimensions through the development of adaptive control strategies and integrating learning-based mechanisms for dynamic parameter adjustment. The algorithm will also be hybridized with machine learning techniques to facilitate automated tuning and problem-specific adaptation. Medium-term objectives include extending the algorithm to multi-objective optimization scenarios, enabling real-time adaptation to dynamic problems with time-varying objectives, and exploring quantum-enhanced variants that utilize quantum parallelism for faster convergence. Additionally, enhancing the exploratory capacity through opposition-based learning and comprehensive learning strategies will be considered. Long-term research will target applications in engineering design, structural optimization, renewable energy, deep learning hyperparameter tuning, neural architecture search, and large-scale industrial systems such as smart grids and supply chain management. These research directions collectively aim to establish EBMLO-DBO as a powerful and adaptable optimization framework suitable for diverse and increasingly complex real-world challenges.

## Supplementary Information

Supplementary Information.

## Data Availability

All data generated or analyzed during this study are included directly in the text of this submitted manuscript. There are no additional external files with datasets.
