# Supplementary material for: Elite Bernoulli-based mutated dung beetle algorithm for global complex problems and parameter estimation of solar photovoltaic models
Source: Sci Rep. 2025 Oct 17;15:36304. doi: 10.1038/s41598-025-06108-4 (PMC12534494; doi:10.1038/s41598-025-06108-4)
Supplement: Supplementary file 1 — Supplementary Information. [file 41598_2025_6108_MOESM1_ESM.docx]

Appendix

Table A1 Statistical analysis based on Wilcoxon test between EBMLO-DBO and its rivals using CEC’17, D=50

| F | **DBO** | **CMAES** | **IMODE** | **AGSK** | **DAOA** | **SCADE** | **RLTLBO** |
| --- | --- | --- | --- | --- | --- | --- | --- |
| C17-G1 | 1.734398LEL-06 | 1.734398LEL-06 | 1.734398LEL-06 | 1.734398LEL-06 | 1.734398LEL-06 | 1.734398LEL-06 | 1.734398LEL-06 |
| C17-G3 | 1.734398LEL-06 | 9.271025LEL-03 | 6.319757LEL-05 | 1.254382LEL-01 | 1.734398LEL-06 | 1.483928LEL-03 | 1.734398LEL-06 |
| C17-G4 | 1.734398LEL-06 | 1.734398LEL-06 | 1.734398LEL-06 | 1.734398LEL-06 | 1.734398LEL-06 | 6.835856LEL-03 | 1.734398LEL-06 |
| C17-G5 | 1.734398LEL-06 | 1.734398LEL-06 | 1.734398LEL-06 | 1.734398LEL-06 | 1.734398LEL-06 | 1.382036LEL-03 | 1.734398LEL-06 |
| C17-G6 | 1.734398LEL-06 | 1.734398LEL-06 | 1.734398LEL-06 | 1.734398LEL-06 | 1.734398LEL-06 | 1.734398LEL-06 | 1.734398LEL-06 |
| C17-G7 | 1.734398LEL-06 | 4.285686LEL-06 | 1.734398LEL-06 | 6.339136LEL-06 | 1.254382LEL-01 | 2.613431LEL-04 | 1.734398LEL-06 |
| C17-G8 | 1.734398LEL-06 | 1.920921LEL-06 | 1.126540LEL-05 | 2.353421LEL-06 | 1.734398LEL-06 | 1.964581LEL-03 | 1.734398LEL-06 |
| C17-G9 | 1.734398LEL-06 | 3.882182LEL-06 | 1.734398LEL-06 | 1.972948LEL-05 | 2.126636LEL-06 | 2.584559LEL-03 | 1.734398LEL-06 |
| C17-G10 | 1.734398LEL-06 | 1.493564LEL-05 | 1.286631LEL-03 | 2.596713LEL-05 | 3.609433LEL-03 | 8.466082LEL-06 | 1.734398LEL-06 |
| C17-G11 | 1.734398LEL-06 | 1.890972LEL-04 | 1.734398LEL-06 | 1.238080LEL-05 | 3.872303LEL-03 | 1.734398LEL-06 | 1.734398LEL-06 |
| C17-G12 | 1.734398LEL-06 | 1.734398LEL-06 | 1.734398LEL-06 | 4.285686LEL-06 | 1.734398LEL-06 | 1.734398LEL-06 | 1.734398LEL-06 |
| C17-G13 | 1.734398LEL-06 | 1.734398LEL-06 | 1.734398LEL-06 | 1.734398LEL-06 | 5.709650LEL-02 | 1.734398LEL-06 | 1.734398LEL-06 |
| C17-G14 | 2.603328LEL-06 | 4.729202LEL-06 | 3.181679LEL-06 | 4.729202LEL-06 | 4.729202LEL-06 | 7.970983LEL-01 | 1.734398LEL-06 |
| C17-G15 | 1.734398LEL-06 | 1.734398LEL-06 | 1.734398LEL-06 | 2.126636LEL-06 | 1.734398LEL-06 | 3.064999LEL-04 | 1.734398LEL-06 |
| C17-G16 | 1.734398LEL-06 | 2.848596LEL-02 | 5.306992LEL-05 | 1.319417LEL-02 | 5.751653LEL-06 | 1.890972LEL-04 | 1.734398LEL-06 |
| C17-G17 | 1.734398LEL-06 | 1.920921LEL-06 | 1.734398LEL-06 | 1.734398LEL-06 | 1.920921LEL-06 | 1.734398LEL-06 | 1.734398LEL-06 |
| C17-G18 | 1.734398LEL-06 | 1.734398LEL-06 | 1.734398LEL-06 | 2.603328LEL-06 | 1.734398LEL-06 | 2.126636LEL-06 | 1.734398LEL-06 |
| C17-G19 | 1.734398LEL-06 | 2.603328LEL-06 | 1.734398LEL-06 | 1.734398LEL-06 | 6.583305LEL-04 | 1.742281LEL-04 | 1.734398LEL-06 |
| C17-G20 | 1.734398LEL-06 | 2.414704LEL-03 | 5.792446LEL-05 | 3.854236LEL-03 | 3.600388LEL-01 | 6.268281LEL-02 | 1.734398LEL-06 |
| C17-G21 | 1.734398LEL-06 | 3.112315LEL-05 | 1.238080LEL-05 | 2.224827LEL-04 | 1.149922LEL-04 | 2.105260LEL-03 | 2.126636LEL-06 |
| C17-G22 | 1.734398LEL-06 | 1.734398LEL-06 | 1.920921LEL-06 | 1.734398LEL-06 | 8.220647LEL-02 | 1.734398LEL-06 | 1.734398LEL-06 |
| C17-G23 | 1.734398LEL-06 | 7.513662LEL-05 | 1.734398LEL-06 | 2.613431LEL-04 | 8.466082LEL-06 | 2.957462LEL-03 | 1.734398LEL-06 |
| C17-G24 | 1.734398LEL-06 | 1.734398LEL-06 | 1.734398LEL-06 | 1.734398LEL-06 | 1.734398LEL-06 | 1.734398LEL-06 | 1.734398LEL-06 |
| C17-G25 | 1.734398LEL-06 | 1.734398LEL-06 | 1.734398LEL-06 | 1.734398LEL-06 | 1.734398LEL-06 | 2.613431LEL-04 | 1.734398LEL-06 |
| C17-G26 | 1.734398LEL-06 | 1.734398LEL-06 | 1.734398LEL-06 | 1.734398LEL-06 | 6.319757LEL-05 | 1.846219LEL-01 | 1.734398LEL-06 |
| C17-G27 | 1.734398LEL-06 | 4.071512LEL-05 | 6.983783LEL-06 | 1.734398LEL-06 | 1.734398LEL-06 | 7.521331LEL-02 | 1.734398LEL-06 |
| C17-G28 | 1.734398LEL-06 | 1.024633LEL-05 | 3.493456LEL-01 | 1.020107LEL-01 | 1.493564LEL-05 | 6.339136LEL-06 | 1.126540LEL-05 |
| C17-G29 | 1.734398LEL-06 | 2.596713LEL-05 | 1.734398LEL-06 | 1.734398LEL-06 | 1.734398LEL-06 | 1.986102LEL-01 | 1.734398LEL-06 |
| C17-G30 | 1.734398LEL-06 | 3.181679LEL-06 | 3.181679LEL-06 | 1.734398LEL-06 | 1.734398LEL-06 | 7.655193LEL-01 | 1.734398LEL-06 |
